# Secondary Metabolites of *Aeromonas veronii* Strain A134 Isolated from a *Microcystis aeruginosa* Bloom

**DOI:** 10.3390/metabo9060110

**Published:** 2019-06-09

**Authors:** Gad Weiss, Dimitry Kovalerchick, Omer Murik, Assaf Sukenik, Aaron Kaplan, Shmuel Carmeli

**Affiliations:** 1 Plants and Environmental Sciences, the Hebrew University of Jerusalem, Edmond J. Safra Campus, Givat Ram, Jerusalem 9190401, Israel; gad.weiss@mail.huji.ac.il (G.W.); omer.murik@mail.huji.ac.il (O.M.); aaron.kaplan@mail.huji.ac.il (A.K.); 2 Raymond and Beverly Sackler School of Chemistry and Faculty of Exact Sciences, Tel Aviv University, Tel Aviv 69978, Israel; Dimitri.Kovalerchik@metabomed.com; 3 Metabomed Ltd., Yavne 81220, Israel; 4 The Yigal Allon Kinneret Limnological Laboratory, Israel Oceanographic and Limnological Research, Migdal 14950, Israel; assaf@ocean.org.il

**Keywords:** *Aeromonas veronii*, *Microcystis aeruginosa*, secondary metabolites

## Abstract

*Aeromonas veronii* strain A134 was isolated from *Microcystis aeruginosa* colonies collected from Lake Kinneret (Sea of Galilee), Israel. The *Aeromonas* culture media inhibited the growth of *M. aeruginosa* (strain MGK). The crude extract of a large-scale culture of *A. veronii* A134 was separated in a few chromatographic steps to yield three new secondary metabolites, 9-chlorolumichrome (**1**), veronimide (**2**) and veronipyrazine (**3**), along with a known lumichrome and several known diketopiperazines. The structures of the new compounds were established by analyses of the data from 1D and 2D NMR experiments and HRMS data of the compounds, as well as a single-crystal X-ray analysis of synthetic **1**. The structure elucidation and proposed biogenesis of the new compounds are described below.

## 1. Introduction

**Figure 1:**
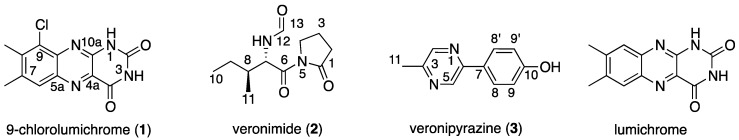
The new metabolites that were isolated from *Aeromonas veronii* strain A134.

Studies on the gram-negative bacteria *Aeromonas* species, which are ubiquitous worldwide in water bodies, have mostly focused on pathogen species associated with fish and human infections [1]. However, very few studies have investigated the biosynthetic capacity of this group of microorganisms. The presence of secondary metabolites has been recognized in various *Aeromonas* species [2] such as *Aeromonas hydrophila*, in which GC-MS analysis suggests the presence of 23 antifungal bioactive compounds [3]. *Aeromonas salmonicida* subsp. *achromogenes* strain NY4, on the other hand, exhibits the capacity to oxidize phenanthrene and to produce a large number of resulting degradation products [4]. In a recent study we showed interspecies interactions between the non-pathogenic *Aeromonas veronii* and a toxic cyanobacterium in a fresh body lake, implicating secondary metabolites in the cross-talk between these organisms. Enhanced lumichrome production by *A. veronii* in response to the presence of culture media from the toxic cyanobacterium *Microcystis aeruginosa* strain MGK and inhibition of MGK growth by applications of pure lumichrome implicated this metabolite in the cross-talk between these two microorganisms [5]. Analysis of its genome (accession no. PRJNA508461) using AntiSMASH [6] identified genes potentially encoding two unknown bacteriocins (ctg42_4, location: 1838–2506; ctg86_35, location: 34489–35346) and a gene cluster likely encoding a non-ribosomal peptide synthetase (ctg90_3, location: 1480–8956). Our present study aimed to identify novel secondary metabolites from *A. veronii* strain A134, which yielded, among others, three new metabolites—9-chlorolumichrome (**1**), veronimide (**2**) and veronipyrazine (**3**) (Figure 1). The structure elucidation of the new metabolites and their possible biogenesis are discussed below.

## 2. Results

### 2.1. Structure Elucidation of 9-Chlorolumichrome (***1***)

9-Chlorolumichrome (**1**) was isolated as a yellowish solid material, which presented high-resolution (HR) negative ESI-MS mass signals ([M−H]^−^) at *m*/*z* 275.0356 and 277.0341 in a 3:1 ion intensity ratio, corresponding to the molecular formula C_12_H_8_N_4_O_2_Cl and 10 degrees of unsaturation. The ^1^H NMR spectrum (Table 1) of **1** resembled that of lumichrome [7] except for the absence of one singlet aromatic proton and small differences in the chemical shifts of its other proton signals when compared with those of lumichrome [8]. In the ^13^C NMR spectrum (Table 1) of **1**, small changes in the chemical shifts of the carbons relative to those of lumichrome and an additional quaternary sp^2^ carbon instead of one of the sp^2^ methine carbons of lumichrome were observed. The latter data suggest that one of the aromatic methines (C-6 or C-9) of lumichrome was substituted by a chlorine atom. Analysis of the 2D NMR spectra of **1** and comparison of the chemical shifts of C-2, C-4 and C-10a with those of lumichrome established **1** as 9-chlorolumichrome. The chlorination of lumichrome with *N*-chlorosuccinimide using the procedure of Tanemura et al. [9] afforded a single product which presented NMR and MS data identical with those of **1**. X-ray analysis of a crystal obtained from the synthetic material confirmed its structure as 9-chlorolumichrome (Figure S1).

### 2.2. Structure Elucidation of Veronimide (***2***)

Veronimide (**2**) was isolated as an amorphous solid that presented high-resolution ESI-MS mass signals ([M+Na]^+^) at *m*/*z* 249.1211 corresponding to the molecular formula C_11_H_18_N_2_O_3_Na and 4 degrees of unsaturation. In the ^1^H NMR spectrum (Table 2), **2** presented, among others, protons attributed to an amide (8.26 ppm), a formyl (8.03 ppm), a down field shifted methine (5.62 ppm), symmetric methylenes (3.66, 2.58, 1.95 ppm), a doublet methyl (0.86 ppm) and a triplet methyl (0.79 ppm). The ^13^C NMR spectrum of **2** revealed, among others, three carboxy carbons (175.7, 172.6, 161.3 ppm), an amino-methine (54.5 ppm) and an amino-methylene (45.7 ppm). Analysis of the COSY spectrum (Table 2) of **2** established two spin systems: methylenes 2 through 4 and NH-12 through CH_3_-11. These two spin systems could be extended through the HSQC and HMBC correlations (Table 2) to pyrrolidinonyl and *N*-formyl-isoleucyl sub-structures. To fulfil the molecular formula of **2**, the latter two sub-structures could be connected only through the carboxyl group of the *N*-formyl-isoleucyl to the pyrrolidinonyl-nitrogen. The absolute stereochemistry of the chiral center of the isoleucine unit was established as L by Marfey’s analysis [10], establishing the structure of veronimide for **2**.

### 2.3. Structure Elucidation of Veronipyrazine (***3***)

Veronipyrazine (**3**) was isolated as an amorphous white solid exhibiting negatively charged HRESI-MS mass signals ([M−H]^−^) at *m*/*z* 185.0718, which corresponded to the molecular formula C_11_H_9_N_2_O and 8 degrees of unsaturation. The ^1^H NMR spectrum of **3** (Table 3) presented three singlet proton signals at the lower field of the spectrum (9.83, 8.97, 8.50 ppm), two doublet signals, 2H each, in the aromatic region of the spectrum (6.87, 7.93 ppm) and a singlet methyl signal resonating at 2.48 ppm. The ^13^CNMR spectrum of **3** (Table 3) presented eight aromatic signals, two of which doubled and a single methyl signal at the aliphatic region. The two aromatic doublet proton signals, 7.93 and 6.87 ppm (2H each), presented mutual COSY correlations suggesting a conjugated phenol ring. The remaining three singlet signals presented very weak COSY correlations of the signal at 8.50 ppm with those at 8.97 and 2.48 ppm, suggesting that they belong to the same aromatic spin system but are coupled through four or more bonds. The chemical shifts of the protons and carbons (Table 3), as well as the C–H coupling constants (obtained from the HSQC experiment), suggested that they belong to an heteroaromatic spin system. The heteroaromatic methine-2 presented HMBC correlation with the aromatic methyl-11. The latter methyl presented an additional HMBC correlation with an heteroaromatic quaternary imine carbon-3, suggesting a direct connection between them. The HMBC correlations of H-2 with C-3, C-5 and C-11, and of H-5 with C-3 and C-6, established the 1,4-pyrazine ring system. Finally, the HMBC correlation of H-8 and H-8’ with C-6 proved the connection of the phenol ring to the pyrazine ring through C-6, establishing structure of veronipyrazine for **3**.

## 3. Discussion

**Scheme 1:**
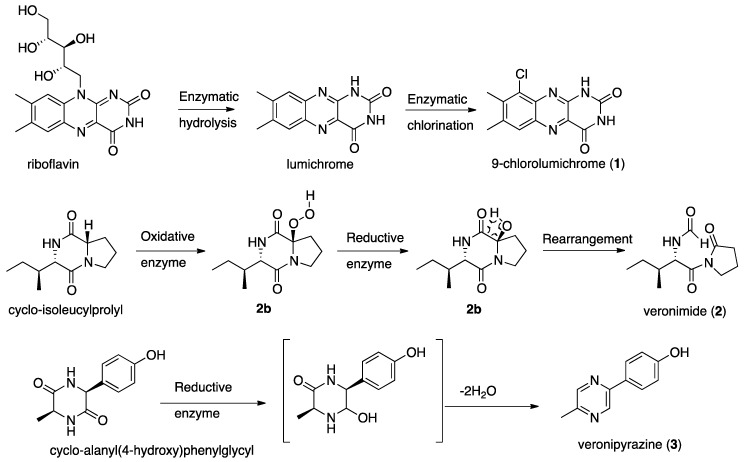
Possible transformations leading to compounds **1**–**3**.

Since the metabolome of *A. veronii* has not been previously studied, we decided to separate and characterize by NMR and MS those metabolites that were present in its extract in sufficient quantities. In the current research we isolated from the culture medium of *A. veronii* strain A134, which inhibited the growth of *M. aeuroginosa* strain MGK, three new metabolites, 9-chlorolumichrome (**1**), veronimide (**2**) and veronipyrazine (**3**), along with the known lumichrome, indole-3-glyoxylamide, cyclo-tyrosylprolyl [11], cyclo-valylprolyl [12], cyclo-isoleucylprolyl [13], cyclo-phenylalanylprolyl [12] and cyclo-tryptophylprolyl [12]. Due to the minute quantities of **1**–**3**, they were tested only for the growth inhibition of *M. aeuroginosa* strain MGK by cell counting, but they were found to be inactive. The only active metabolite we isolated from the culture medium was lumichrome [5]. The abovementioned metabolites, **1**–**3**, are most probably derived from the enzymatic transformation of primary metabolites rather than products of secondary metabolism by special biosynthetic genes. Lumichrome and 9-chlorolumichrome (**1**) are possibly derived from the hydrolysis of the sugar unit of riboflavin, which is followed by chlorination, probably by a flavin-dependent holgenase [14] (Scheme 1). Veronimide (**2**) seems to be an oxidation product of cyclo-isolecinylprolyl, probably resulting from the action of cytochrome-P450 or a similar oxidizing enzyme, which produces the peroxy-derivative **2a**. Intermediate **2a** is most probably converted to the corresponding alcohol **2b**, which in turn is rearranged to veronimide (**2**) (Scheme 1). Veronipyrazine (**3**) is most probably derived from the reduction of one of the amide carboxamides and the subsequent double dehydration of cyclo-alanyl(4-hydroxy)phenylglycyl (Scheme 1). These findings for *A. veronii* strain A134, and those published for *A. salmonicida* subsp. *achromogenes* strain NY4 [4], suggest that *Aeromonas* spp. are capable of transforming primary metabolites to secondary-like metabolites but have very low capacity to produce secondary metabolites. How and whether these abilities are associated with their pathogenesis deserves further investigation.

## 4. Materials and Methods 

### 4.1. General Experimental Procedures

Optical rotation values were obtained on a JASCO P-1010 polarimeter at the sodium D line (589 nm). UV spectra were recorded on an Agilent 8453 spectrophotometer. IR spectra were recorded on a Bruker Tensor 27 FT-IR instrument. NMR spectra were recorded on Bruker Avance and Avance III spectrometers at 500.13 MHz for ^1^H and 125.76 MHz for ^13^C. DEPT, COSY-45, gTOCSY, gROESY, gHSQC, gHMQC and gHMBC spectra were recorded using standard Bruker pulse sequences. NMR chemical shifts were referenced to TMS δ_H_ and δ_C_ = 0 ppm. High-resolution mass spectra were recorded on a Waters MaldiSynapt instrument (ESI) and LC-MS spectra were recorded on a Waters Xevo TQD instrument (ESI). HPLC separations were performed on a Merck Hitachi HPLC system (L-6200 Intelligent pump and L-4200 UV-VIS detector), a JASCO P4-2080 plus HPLC system with a multiwavelength detector and an Agilent 1100 Series HPLC system.

### 4.2. Isolation and Identification of A. veronii Strain A134 

Water samples were collected from 52 *Microcystis* sp. surface scums at various sites in Lake Kinneret (Sea of Galilee), Israel, during a bloom event in February 2014. Aliquots of 100 µL were placed on 1.5% agar petri dishes containing Luria–Bertani (LB) medium and incubated at 30 °C. Single colonies were isolated and sub-cultured by streaking onto solid or into liquid LB; the latter were agitated (100 rpm) at 30 °C. Those isolates that were able to grow on polysaccharides isolated from *Microcystis* sp. were identified using their 16S rDNA sequences. DNA was isolated from LB grown cultures using phenol and chloroform by a standard method [15]. Sequencing of the 16S rDNA was performed by an ABI PRISM 3730xl DNA Analyzer [16]. The forward (F) and reverse (R) primers used were: F-AGAGTTTGATCMTGGCTCAG and R-TACGGYTACCTTGTTACGACTT. Analysis of the 16S rDNA sequence of strain A134 clearly indicated that the isolated bacterium was *A. veronii* (see phylogenetic tree in Figure S1 in the Supplementary Material).

### 4.3. Isolation and Identification of the Metabolites

*A. veronii* strain A134 cultures were grown in 20 L LB medium for two weeks and the cells were removed from the medium by centrifugation. The spent medium was freeze-dried and extracted with a 45:45:10 mixture of ethyl acetate/methanol/water yielding 28 g of crude extract. After each step of separation, ^1^H-NMR spectra and biological activity assays were performed in order to guide further separations. The extract was partitioned successively between petroleum ether, a mixture of 1:1 chloroform/methanol and water. The chloroform/methanol soluble material (18.5 gr) was separated on a reversed-phase C-18 open column eluted with a step-gradient of decreasing polarity, from 100% water to 100% methanol and 100% ethyl acetate. Fractions 5–9, which inhibited *M. aeuroginosa* strain MGK growth, were combined (1.2 gr) and further separated on a size exclusion Sephadex LH-20 column eluted with a 1:1 mixture of chloroform/methanol to afford two active fractions (9 and 10, 33.8 mg). The combined active fraction was separated on a preparative Phenomenex Hydro-RP HPLC column eluted with an isocratic solvent mixture of 2:1 0.1% aqueous TFA/CH_3_CN to afford in fraction 6 (t_R_ 21.3 min, 1.5 mg, 0.005% yield of dry crude extract weight) lumichrome and in fraction 11 (t_R_ 39.0 min, 0.4 mg, 0.001%) yield 9-chlorolumichrome (**1**). The combined non-active fractions (3–8, 0.42 g) from the size exclusion column were further separated on a Cosmosyl C-8 HPLC column eluted with 3:1 0.1% aqueous TFA/CH_3_CN to afford in fraction 2, cyclo-tyrosylprolyl (t_R_ 14.8 min, 9.8 mg, 0.033% yield), in fraction 3, cyclo-valylproly (t_R_ 15.8 min, 12.8 mg, 0.043% yield), in fraction 5, cyclo-isoleucylprolyl (t_R_ 18.4 min, 8.4 mg, 0.028% yield), in fraction 9, cyclo-phenylalanylprolyl (t_R_ 26.0 min, 79.6 mg, 0.265% yield) and in fraction 11, cyclo-tryptophylprolyl (t_R_ 18.4 min, 7.3 mg, 0.024% yield). Fraction 6 (50.2 mg) and fraction 13 (16.1 mg) from the latter separation were further separated on the same column eluted with 45:55 MeOH/Water. Fraction 6 yielded in fraction 3, pure **2** (t_R_ 27.4 min, 1.4 mg, 0.005% yield), while fraction 13 yielded in fraction 1, indole-3-glyoxylamide (t_R_ 28.8 min, 3.4 mg, 0.011% yield) and in fraction 3, pure **3** (t_R_ 38.3 min, 1.2 mg, 0.004% yield).

9-Chlorolumichrome (**1**): Crystalline yellowish solid (monoclinic, isopropanol); UV (MeOH) λ_max_ (log ε) 221 (4.49) nm, 252 (4.44) nm, 267 (4.50) nm, 349 (3.97) nm, 391 (3.82) nm; IR (ATR Diamond) ν_max_ 3709, 3691, 3648, 3606, 2360, 2341, 2273 cm^−1^; For ^1^H and ^13^C NMR data see Table 1; HRESIMS *m*/*z* 275.0356 [M-H]^−^ (Calcd. for C_12_H_8_N_4_O_2_^35^Cl, 275.0336).

Veronimide (**2**): Amorphous white solid; [α]^25^_D_ 26.7 (*c* 0.15, MeOH); UV (MeOH) λ_max_ (log ε) 215 (3.70) nm; IR (ATR Diamond) ν_max_ 3308, 2966, 2934, 2878, 2273, 1740, 1670 cm^−1^; For ^1^H and ^13^C NMR data see Table 2; HRESIMS *m*/*z* 249.1211 [M+Na]^+^ (Calcd. for C_11_H_18_N_2_O_3_Na, 249.1215). Retention times of the L-FDAA Marfey’s derivatives: L-Ile 49.0 min (D-Ile 53.2 min). 

Veronipyrazine (**3**): Amorphous white solid; UV (MeOH) λ_max_ (log ε) 202 (3.46), 243 (3.11), 269 (3.24), 290 (3.23), 325 (3.04) nm; IR (ATR Diamond) ν_max_ 3212, 1670, 1636, 1611 cm^−1^; For ^1^H and ^13^C NMR data see Table 3; HRESIMS *m*/*z* 185.0718 ([M−H]^−^) (Calcd. for C_11_H_9_N_2_O, 185.0715).

### 4.4. Synthesis of 9-Chlorolumichrome

Lumichrome 50 mg (0.207 mmol), *N*-chlorosuccinimide 55 mg (0.412 mmol) and FeCl_3_ anhydrous 3 mg (0.019 mmol) dissolved in DMF (10 mL) were stirred at rt for 5 days. The solvent was removed under reduced pressure and the residue was purified by column chromatography on silica gel, with a 10% step gradient from CHCl_3_ to MeOH, affording in fraction 1, 9-chlorolumichrome (23 mg, 0.098 mmol, 47% yield).

### 4.5. Determination of the Absolute Configuration of the Amino Acids 

Compound **2** (0.25 mg) was hydrolyzed in 6N, HCl (1 mL). The reaction mixture was maintained in a sealed glass bomb at 104 °C for 16 h. The acid was removed in vacuo and the residue was re-suspended in 250 µL of water. FDAA solution ((1-fluoro-2,4-dinitrophenyl)-5-L-alanine amide) in acetone (115 µL, 0.03 M) and NaHCO_3_ (120 µL, 1M) was added to the reaction vessel. The reaction mixture was stirred at 40 °C for 2 h. Then HCl (2M, 60 µL) was added to each reaction vessel and the solution was evaporated in vacuo. The FDAA-amino acid derivatives from the hydrolysate were dissolved in 1 mL CH_3_CN and compared with standard FDAA-amino acids by HPLC analysis: LiChrospher 60, RP-select B (5 µm), flow rate 1 mL/min, UV detection at 340 nm, linear gradient elution from 9:1 0.1% aq. TFA buffer, pH 3: CH_3_CN to CH_3_CN, within 60 min. The absolute configuration of each amino acid was determined by spiking the derivatized hydrolysates with a d,l-mixture of isoleucine.

## Figures and Tables

**Table 1:** NMR data of 9-chlorolumichrome (**1**) and lumichrome in DMSO-*d*_6_^a^.

	9-Chlorolumichrome (1)			Lumichrome	
Position	δ_C_, Mult.^b^	δ_H_, Mult.^c^	LR^d^ C-H Correlations	δ_C_, Mult.^b^	δ_H_, Mult.^c^
1	-	11.74, s^e^	-	-	11.65, s
2	150.2, C	-	-	150.3, C	-
3	-	12.03, s	-	-	11.82, s
4	160.5, C	-	-	160.8, C	-
4a	131.5, C	-	1, 3	130.4, C	-
5a	138.3, C	-	6	138.6, C	-
6	127.9, CH	7.94, s	11	128.9, CH	7.91, s
7	139.0, C	-	6, 11, 12	144.9, C	-
8	141.7, C	-	11, 12	139.1, C	-
9	128.7, C	-	6, 12	126.0, CH	7.70, s
9a	138.2, C	-	6	141.8, C	-
10a	146.9, C	-	-	146.7, C	-
11	21.0, CH_3_	2.52, s	6	19.8, CH_3_	2.45, s
12	17.9, CH_3_	2.57, s	11	20.4, CH_3_	2.48, s

^a^ 500 MHz for ^1^H and 125 MHz for ^13^C. ^b^ Multiplicity and assignment from HSQC experiment. ^c^ Determined by HMBC experiment. ^d^ Long-range. ^e^ Singlet.

**Table 2:** NMR data of veronimide (**2**) in DMSO-*d*_6_^a^.

Position	δ_C_, Mult.^b^	δ_H_, Mult.^c^ (*J* in Hz)	COSY Correlations	LR^d^ C-H Correlations
1	175.7, C	-	-	2a, 2b, 3a, 3b, 4a, 4b
2	33.4, CH_2_	a 2.61, m^e^ b 3.57, m^e^	3a, 3b	4a, 4b
3	16.9, CH_2_	a 1.96, m^e^ b 1.92, m^e^	2a, 2b, 4a, 4b	2a, 2b, 4a, 4b
4	45.7, CH_2_	a 3.69, m^e^ b 3.65, m^e^	3a, 3b	2a, 2b
6	172.6, C	-	-	7
7	54.5, CH	5.62, dd^f^ (9.1, 4.4)	8, 12	12, 13
8	36.6, CH	1.79, dddq^g^ (10.0, 4.4, 3.9, 6.8)	7, 9a, 9b, 11	7, 9a, 9b, 10, 11
9	23.2, CH_2_	a 1.27, ddq^h^ (13.5, 3.9, 7.4) b 1.08, ddq (13.5, 10.0, 7.4)	8, 9b, 10 8, 9a, 10	7, 9a, 9b, 10, 11
10	11.6, CH_3_	0.79, t^i^ (7.4)	9a, 9b	9a, 9b
11	16.1, CH_3_	0.85, d^j^ (6.8)	8	7, 9a, 9b
12	-	8.26, brd^k^ (9.1)	7, 13	-
13	161.3, CH	8.03, s^l^	12	7

^a^ 500 MHz for ^1^H and 125 MHz for ^13^C. ^b^ Multiplicity and assignment from HSQC experiment. ^c^ Determined by HMBC experiment. ^d^ Long-range. ^e^ Second-order multiplet. ^f^ Double-doublet. ^g^ Double-double-double-quartet. ^h^ Double-double-quartet. ^i^ Triplet. ^j^ Doublet. ^k^ Broad-doublet. ^l^ Singlet.

**Table 3:** NMR data of veronipyrazine (**3**) in DMSO-*d*_6_^a^.

Position	δ_C_, Mult.^b^ (*J*_CH_ in Hz)	δ_H_, Mult.^c^ (*J* in Hz)	LR^d^ C-H Correlations
2	143.5, CH (181.4)	8.50, brs^e^	11
3	150.8, C	-	2, 5, 11
5	140.0, CH (185.1)	8.97, s^f^	-
6	149.0, C	-	2, 5, 8, 8’
7	127.0, C	-	9, 9’
8, 8’	128.0, CH × 2	7.92, d^g^ × 2 (8.7)	-
9, 9’	115.9, CH × 2	6.87, d × 2 (8.7)	9’, 9
10	159.1, C	-	8, 8’, 9, 9’, 10-OH
10-OH	-	9.83, brs	-
11	20.9, CH_3_	2.48, s	2

^a^ 500 MHz for ^1^H and 125 MHz for ^13^C. ^b^ Multiplicity and assignment from HSQC experiment. ^c^ Determined by HMBC experiment. ^d^ Long-range. ^e^ Broad singlet. ^f^ Singlet. ^g^ Doublet.

## Supplementary Materials

The following are available online at https://www.mdpi.com/2218-1989/9/6/110/s1, Figure S1. Phylogenetic tree of *Aeromonas veronii* strain A134; Figure S2. ^1^H NMR spectrum of isolated 9-chlorolumichrome (**1**) in DMSO-*d*_6_; Figure S3. Positive and negative ESIMS spectra of isolated 9-chlorolumichrome (**1**); Figure S4. HRESIMS of isolated 9-chlorolumichrome (**1**); Figure S5. ^1^H NMR spectrum of synthetic 9-chlorolumichrome (**1**) in DMSO-*d*_6_; Figure S6. ^13^C NMR spectrum of synthetic 9-chlorolumichrome (**1**) in DMSO-*d*_6_; Figure S7. HSQC spectrum of synthetic 9-chlorolumichrome (**1**) in DMSO-*d*_6_; Figure S8. HMBC spectrum of synthetic 9-chlorolumichrome (**1**) in DMSO-*d*_6_; Figure S9. COSY spectrum of synthetic 9-chlorolumichrome (**1**) in DMSO-*d*_6_; Figure S10. ESIMS of synthetic 9-chlorolumichrome (**1**); Figure S11. ^1^H NMR spectrum of veronimide (**2**) in DMSO-*d*_6_; Figure S12. ^13^C NMR spectrum of veronimide (**2**) in DMSO-*d*_6_; Figure S13. HSQC spectrum of veronimide (**2**) in DMSO-*d*_6_; Figure S14. HMBC spectrum of veronimide (**2**) in DMSO-*d*_6_; Figure S15. COSY spectrum of veronimide (**2**) in DMSO-*d*_6_; Figure S16. HRESIMS of veronimide (**2**); Figure S17. ^1^H NMR spectrum of veronipyrazine (**3**) in DMSO-*d*_6_; Figure S18. ^13^C NMR spectrum of veronipyrazine (**3**) in DMSO-*d*_6_; Figure S19. HSQC spectrum of veronipyrazine (**3**) in DMSO-*d*_6_; Figure S20. HMBC spectrum of veronipyrazine (**3**) in DMSO-*d*_6_; Figure S21. COSY spectrum of veronipyrazine (**3**) in DMSO-*d*_6_; Figure S22. HRESIMS of veronipyrazine (**3**); Figure S23. ^1^H NMR spectrum of indole-3-glyoxylamide in DMSO-*d*_6_; Figure S24. ^13^C NMR spectrum of indole-3-glyoxylamide in DMSO-*d*_6_; Figure S25. HSQC spectrum of indole-3-glyoxylamide in DMSO-*d*_6_; Figure S26. HMBC spectrum of indole-3-glyoxylamide) in DMSO-*d*_6_; Figure S27. COSY spectrum of indole-3-glyoxylamide in DMSO-*d*_6_; Figure S28. ESIMS of indole-3-glyoxylamide; Crystal Structure Report and Table S11: Data collection details for synthetic 9-chlorolumichrome (**1**)**;** Table S2. Sample and crystal data for synthetic 9-chlorolumichrome (**1**); Table S3. Data collection and structure refinement for synthetic 9-chlorolumichrome (**1**); Table S4. Atomic coordinates and equivalent isotropic atomic displacement parameters (Å^2^) for synthetic 9-chlorolumichrome (**1**); Table S5. Atomic coordinates and equivalent isotropic atomic displacement parameters (Å^2^) for synthetic 9-chlorolumichrome (**1**); Table S6. Bond angles (°) and molecular structure for synthetic 9-chlorolumichrome (**1**); Table S7. Torsion angles (°) for synthetic 9-chlorolumichrome (**1**); Table S8. Anisotropic atomic displacement parameters (Å^2^) for synthetic 9-chlorolumichrome (**1**); Table S9. Hydrogen atomic coordinates and isotropic atomic displacement parameters (Å^2^) for synthetic 9-chlorolumichrome (**1**).
