# Supplementary material for: Secondary Metabolites of Aeromonas veronii Strain A134 Isolated from a Microcystis aeruginosa Bloom
Source: Metabolites. 2019 Jun 9;9(6):110. doi: 10.3390/metabo9060110 (PMC6631032; doi:10.3390/metabo9060110)

Secondary metabolites of *Aeromonas veronii* strain A134 isolated from a *Microcystis aeruginosa* bloom

Supplementary Materials

Gad Weiss[1], Dimitry Kovalerchick[2,3], Omer Murik[1], Assaf Sukenik[4], Aaron Kaplan[1] and Shmuel Carmeli[2*]

[1]Plants and Environmental Sciences, the Hebrew University of Jerusalem, Edmond J. Safra Campus, Givat Ram, Jerusalem 9190401, Israel

[2]Raymond and Beverly Sackler School of Chemistry and Faculty of Exact Sciences, Tel Aviv University, Tel Aviv 69978, Israel

[3]Metabomed Ltd, Yavne 81220, Israel

[4]The Yigal Allon Kinneret Limnological Laboratory, Israel Oceanographic and Limnological Research, Migdal, Israel

*Corresponding author: Prof. Shmuel Carmeli, Raymond and Beverly Sackler School of Chemistry and Faculty of Exact Sciences, Tel Aviv University, Tel Aviv 69978, Israel

Email: carmeli@tauex.tau.ac.il, Phone: 972 3 6408450

Table of content

S1. Figure S1. Phylogenetic tree of *Aeromonas veronii* strain A134
S2. Figure S2. ^{1}H NMR spectrum of isolated 9-chlorolumichrome (**1**) in DMSO-d_6
S3. Figure S3. Positive and negative ESIMS spectra of isolated 9-chlorolumichrome (**1**)
S4. Figure S4. HRESIMS of isolated 9-chlorolumichrome (**1**)
S5. Figure S5. ^{1}H NMR spectrum of synthetic 9-chlorolumichrome (**1**) in DMSO-d_6
S6. Figure S6. ^{13}C NMR spectrum of synthetic 9-chlorolumichrome (**1**) in DMSO-d_6
S7. Figure S7. HSQC spectrum of synthetic 9-chlorolumichrome (**1**) in DMSO-d_6
S8. Figure S8. HMBC spectrum of synthetic 9-chlorolumichrome (**1**) in DMSO-d_6
S9. Figure S9. COSY spectrum of synthetic 9-chlorolumichrome (**1**) in DMSO-d_6
S10. Figure S10. ESIMS of synthetic 9-chlorolumichrome (**1**)
S11. Crystal Structure Report and Table S1: Data collection details for synthetic 9-Chlorolumichrome (**1**)
S12. Table S2. Sample and crystal data for synthetic 9-Chlorolumichrome (**1**)
S13. Table S3. Data collection and structure refinement for synthetic 9-Chlorolumichrome (**1**)
S14. Table S4. Atomic coordinates and equivalent isotropic atomic displacement parameters (Å^2) for synthetic 9-Chlorolumichrome (**1**)
S15. Table S5. Atomic coordinates and equivalent isotropic atomic displacement parameters (Å^2) for synthetic 9-Chlorolumichrome (**1**)
S16. Table S6. Bond angles (°) and molecular structure for synthetic 9-Chlorolumichrome (**1**)
S18. Table S7. Torsion angles (°) for synthetic 9-Chlorolumichrome (**1**)
S19. Table S8. Anisotropic atomic displacement parameters (Å^2) for synthetic 9-Chlorolumichrome (**1**)
S20. Table S9. Hydrogen atomic coordinates and isotropic atomic displacement parameters (Å^2) for synthetic 9-Chlorolumichrome (**1**)
S21. Figure S11. ^{1}H NMR spectrum of veronimide (**2**) in DMSO-d_6
S22. Figure S12. ^{13}C NMR spectrum of veronimide (**2**) in DMSO-d_6
S23. Figure S13. HSQC spectrum of veronimide (**2**) in DMSO-d_6
S24. Figure S14. HMBC spectrum of veronimide (**2**) in DMSO-d_6
S25. Figure S15. COSY spectrum of veronimide (**2**) in DMSO-d_6
S26. Figure S16. HRESIMS of veronimide (**2**)
S27. Figure S17. ^{1}H NMR spectrum of veronipyrazine (**3**) in DMSO-d_6
S28. Figure S18. ^{13}C NMR spectrum of veronipyrazine (**3**) in DMSO-d_6
S29. Figure S19. HSQC spectrum of veronipyrazine (**3**) in DMSO-d_6
S30. Figure S20. HMBC spectrum of veronipyrazine (**3**) in DMSO-d_6
S31. Figure S21. COSY spectrum of veronipyrazine (**3**) in DMSO-d_6
S32. Figure S22. HRESIMS of veronipyrazine (**3**)

S33. Figure S23. ^{1}H NMR spectrum of indole-3-glyoxylamide in DMSO-d_6
S34. Figure S24. ^{13}C NMR spectrum of indole-3-glyoxylamide in DMSO-d_6
S35. Figure S25. HSQC spectrum of indole-3-glyoxylamide in DMSO-d_6
S36. Figure S26. HMBC spectrum of indole-3-glyoxylamide) in DMSO-d_6
S37. Figure S27. COSY spectrum of indole-3-glyoxylamide in DMSO-d_6
S38. Figure S28. ESIMS of indole-3-glyoxylamide

S1. Figure S1. Phylogenetic tree of *Aeromonas veronii* strain A134

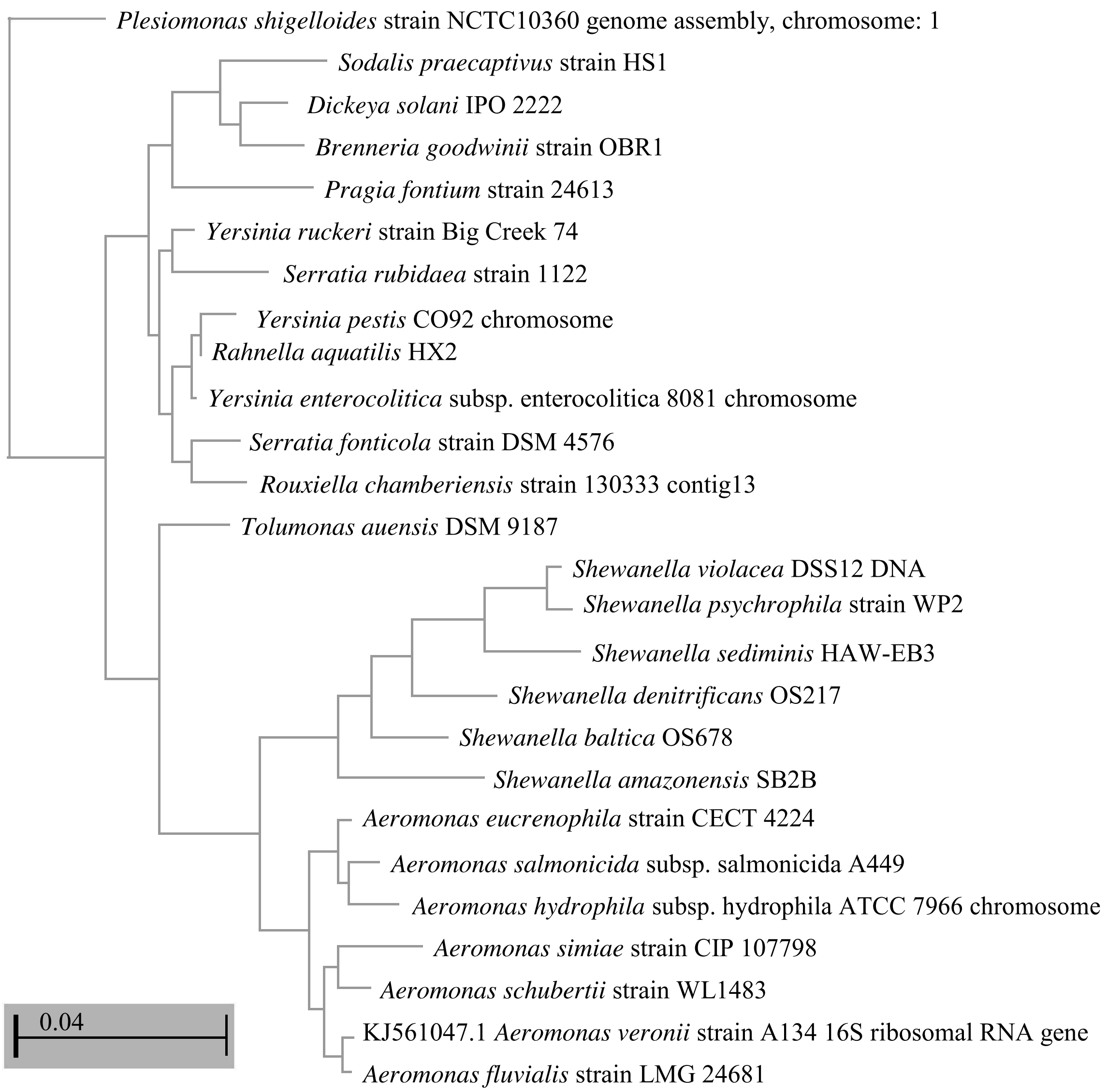

S2. Figure S2. ^{1}H NMR spectrum of isolated 9-Chlorolumichrome (**1**) in DMSO-d_6

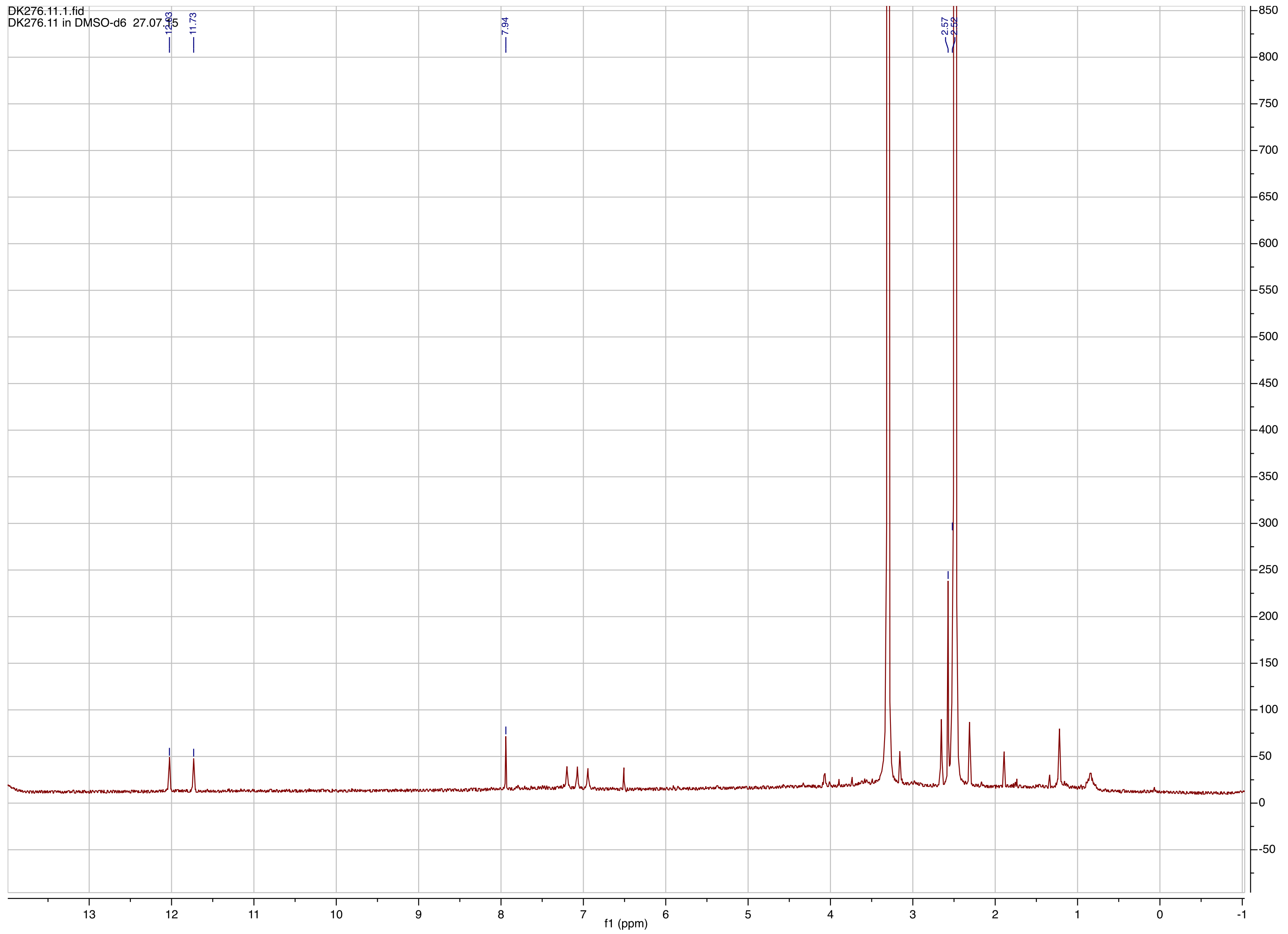

S3. Figure S3. Positive and negative ESIMS spectra of isolated 9-Chlorolumichrome (**1**)

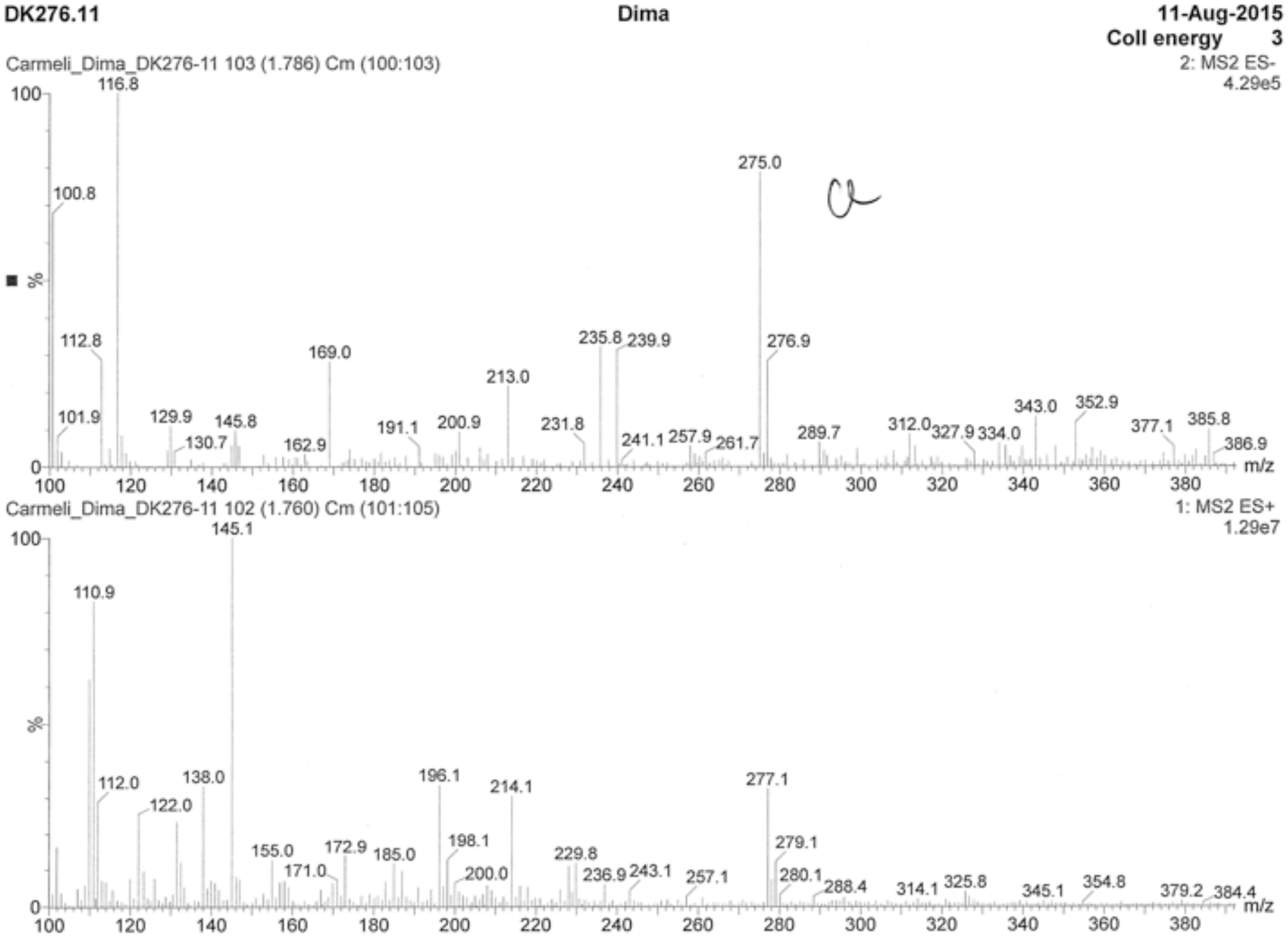

S4. Figure S4. HR ESIMS of isolated 9-Chlorolumichrome (**1**)

Elemental Composition Report — **Page 1**

Single Mass Analysis
Tolerance = 10.0 PPM / DBE: min = -1.5, max = 50.0
Element prediction: Off
Number of isotope peaks used for i-FIT = 3

Monoisotopic Mass, Even Electron Ions
545 formula(e) evaluated with 4 results within limits (all results (up to 1000) for each mass)
Elements Used:
C: 10-15 H: 5-15 N: 0-10 O: 0-10 23Na: 0-1 Cl: 0-2
DK276.11 Dima
carmeli1037b 2 (0.104) Cm (2) 1: TOF MS ES- 8.01e+002

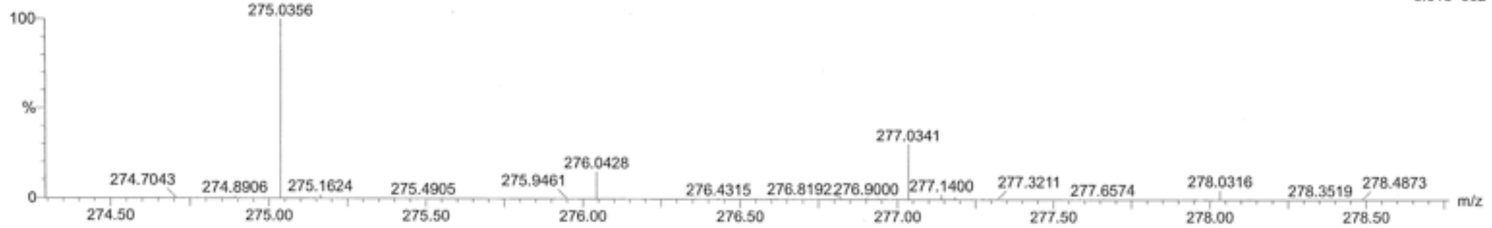

Minimum:				-1.5			
Maximum:		5.0	10.0	50.0			

Mass	Calc. Mass	mDa	PPM	DBE	i-FIT	i-FIT (Norm)	Formula
275.0356	275.0352	0.4	1.5	11.5	54.4	0.4	C15 H9 N2 23Na Cl
	275.0336	2.0	7.3	10.5	55.1	1.1	•C12 H8 N4 O2 Cl
	275.0370	-1.4	-5.1	6.5	60.8	6.9	C14 H14 23Na Cl2
	275.0354	0.2	0.7	5.5	61.3	7.4	C11 H13 N2 O2 Cl2

S5. Figure S5. ^{1}H NMR spectrum of synthetic 9-Chlorolumichrome (**1**) in DMSO-d_6

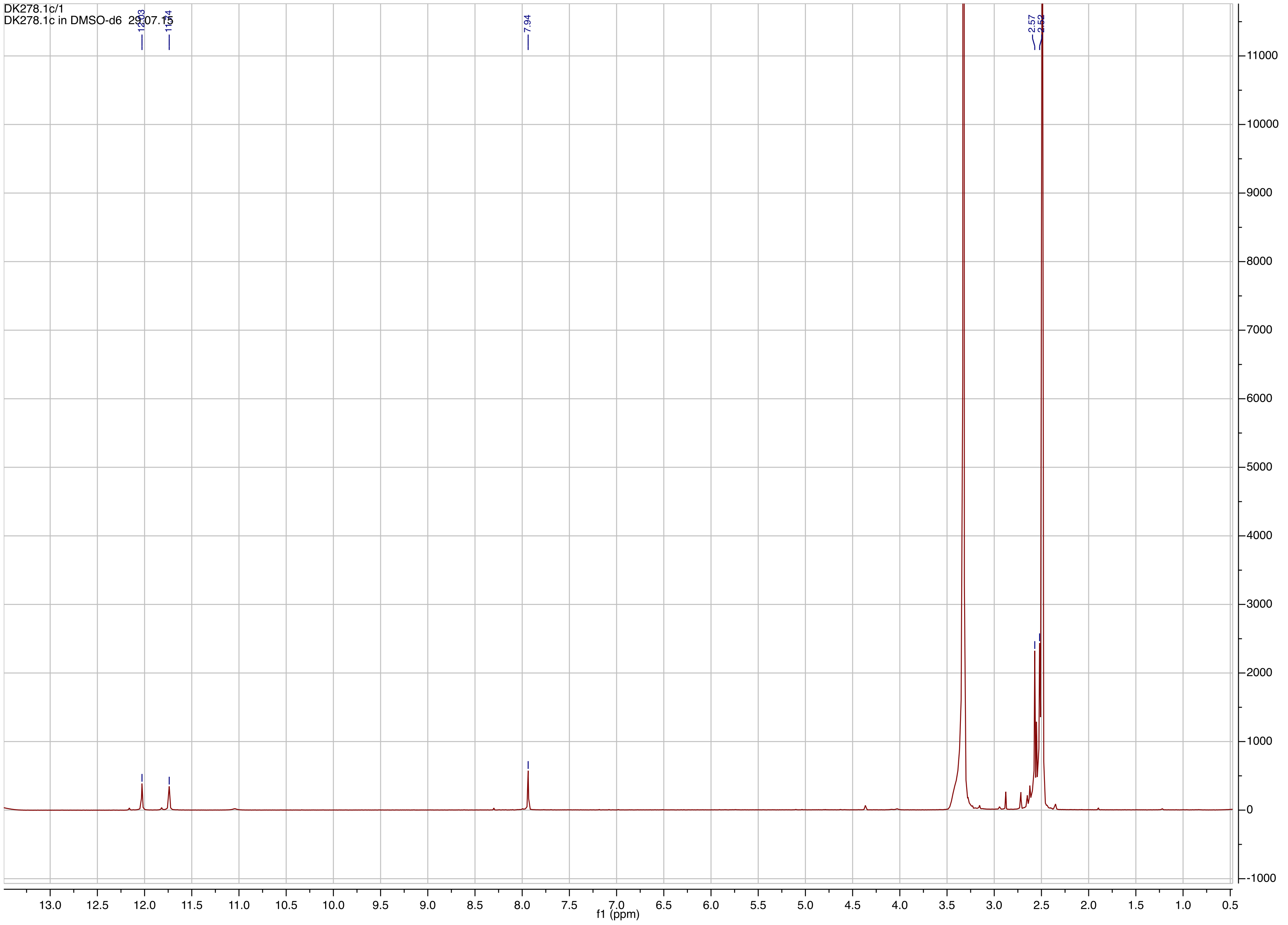

S6. Figure S6. ^{13}C NMR spectrum of synthetic 9-Chlorolumichrome (**1**) in DMSO-d_6

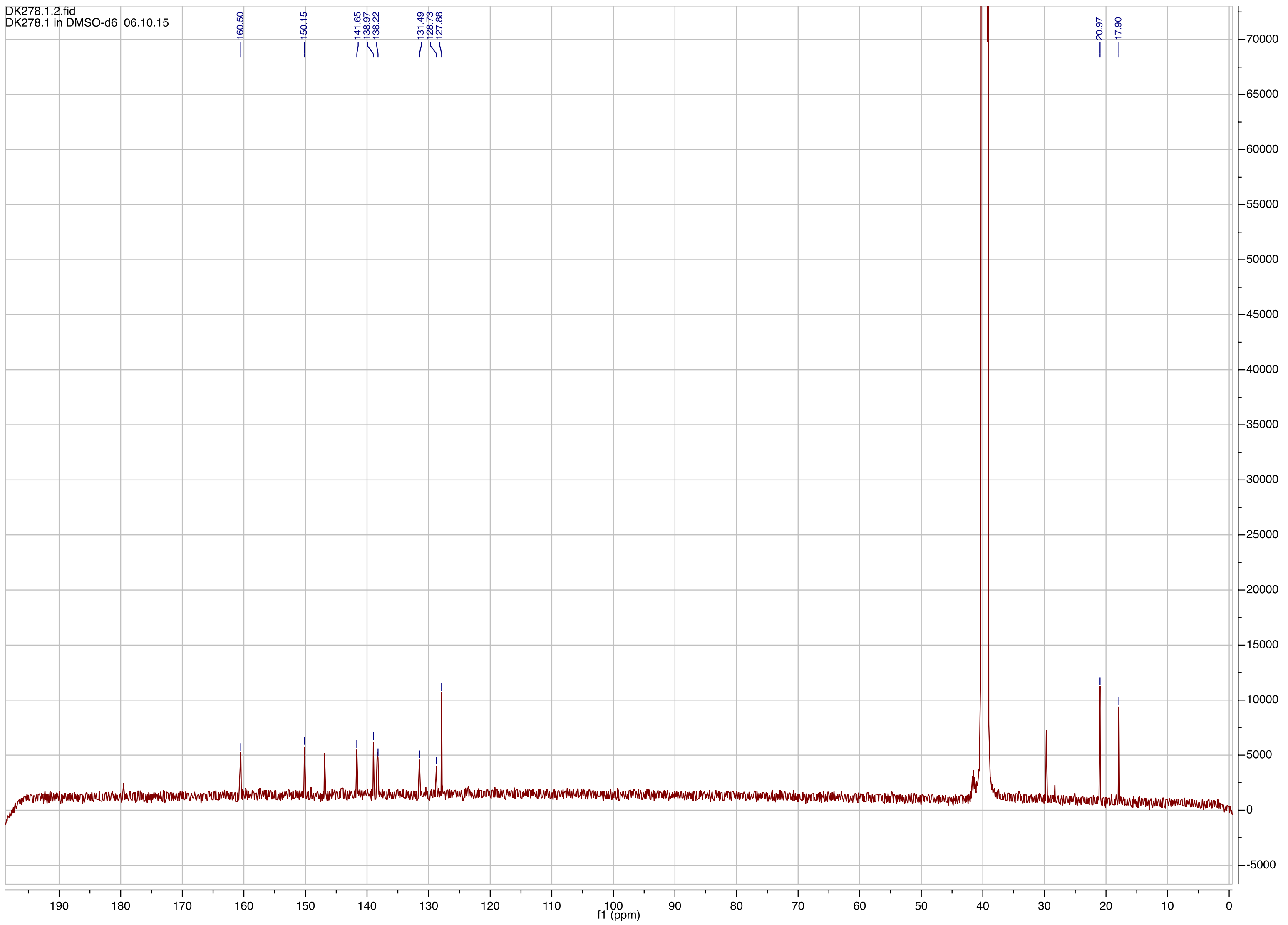

S7. Figure S7. HSQC spectrum of synthetic 9-Chlorolumichrome (**1**) in DMSO-d_6

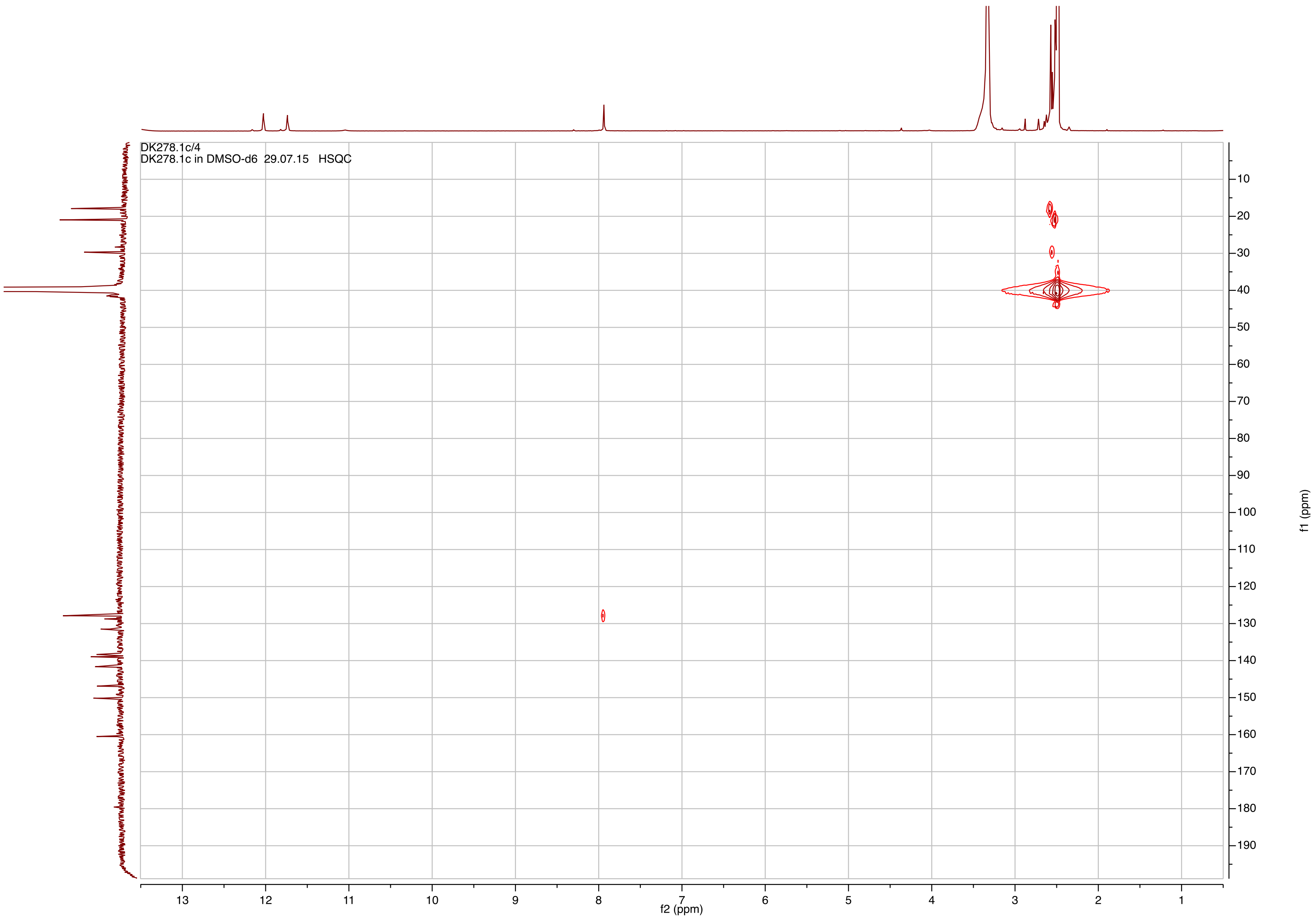

S8. Figure S8. HMBC spectrum of synthetic 9-Chlorolumichrome (**1**) in DMSO-d_6

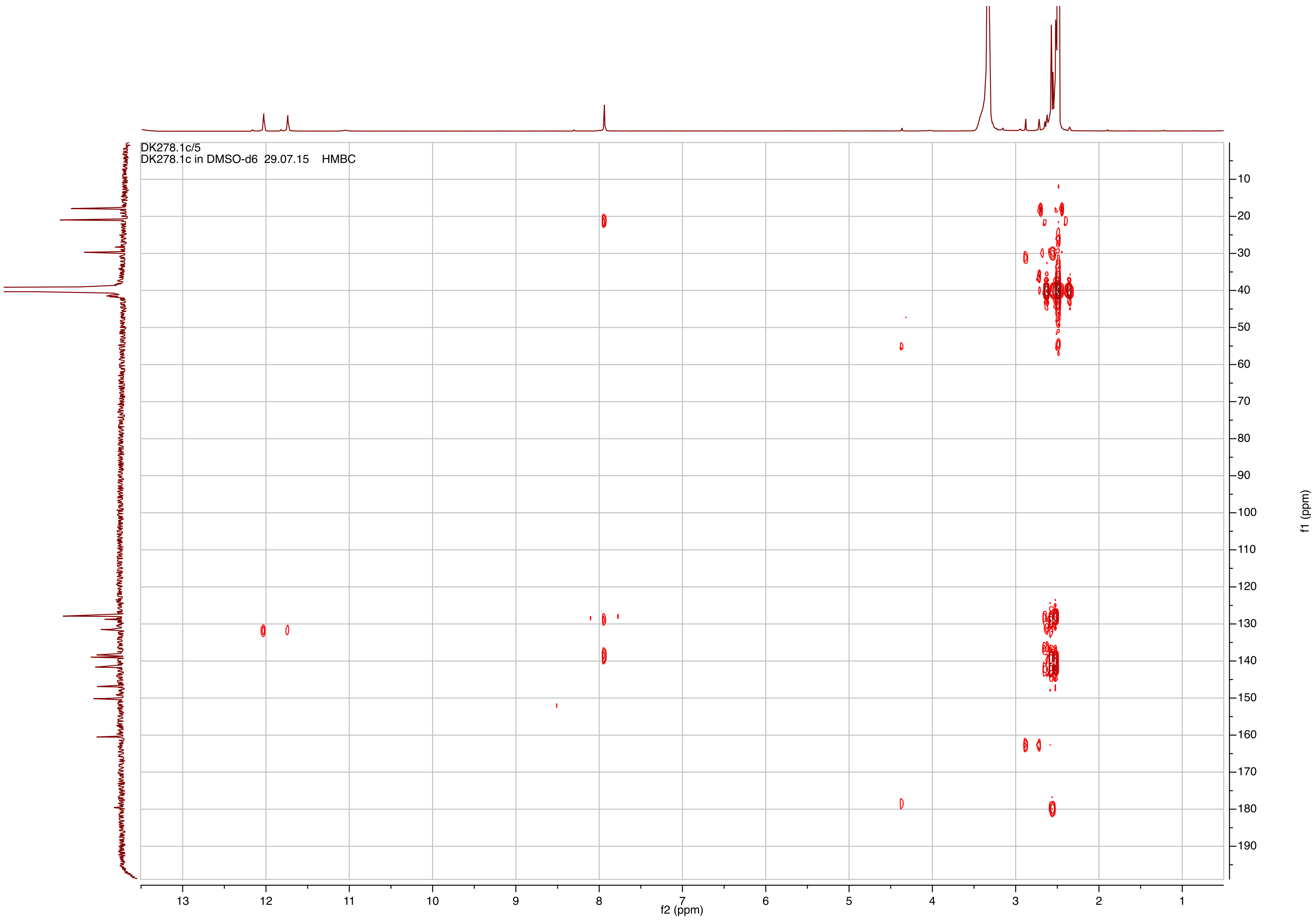

S9. Figure S9. COSY spectrum of synthetic 9-Chlorolumichrome (**1**) in DMSO-d_6

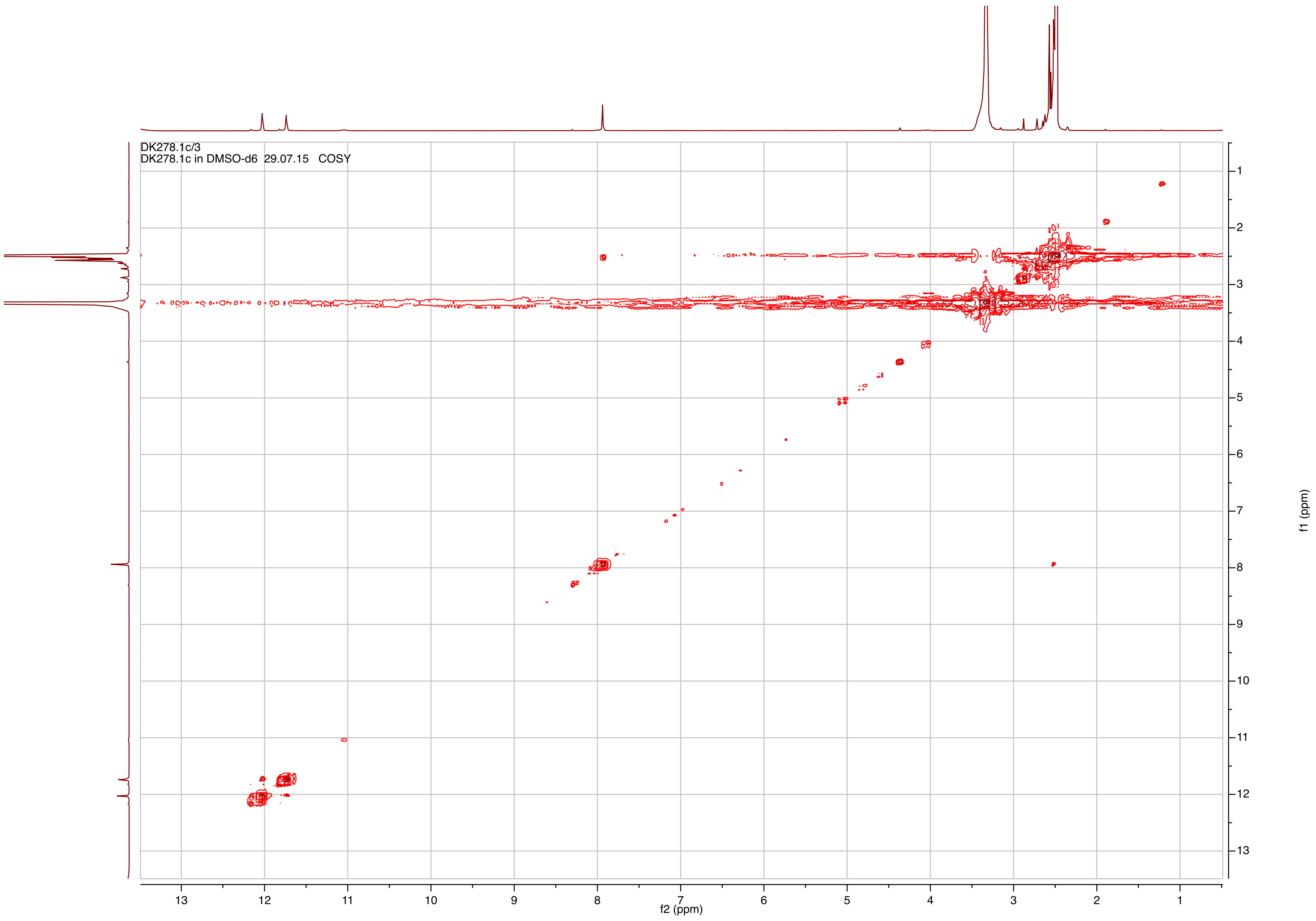

S10. Figure S10. ESIMS of synthetic 9-Chlorolumichrome (**1**)

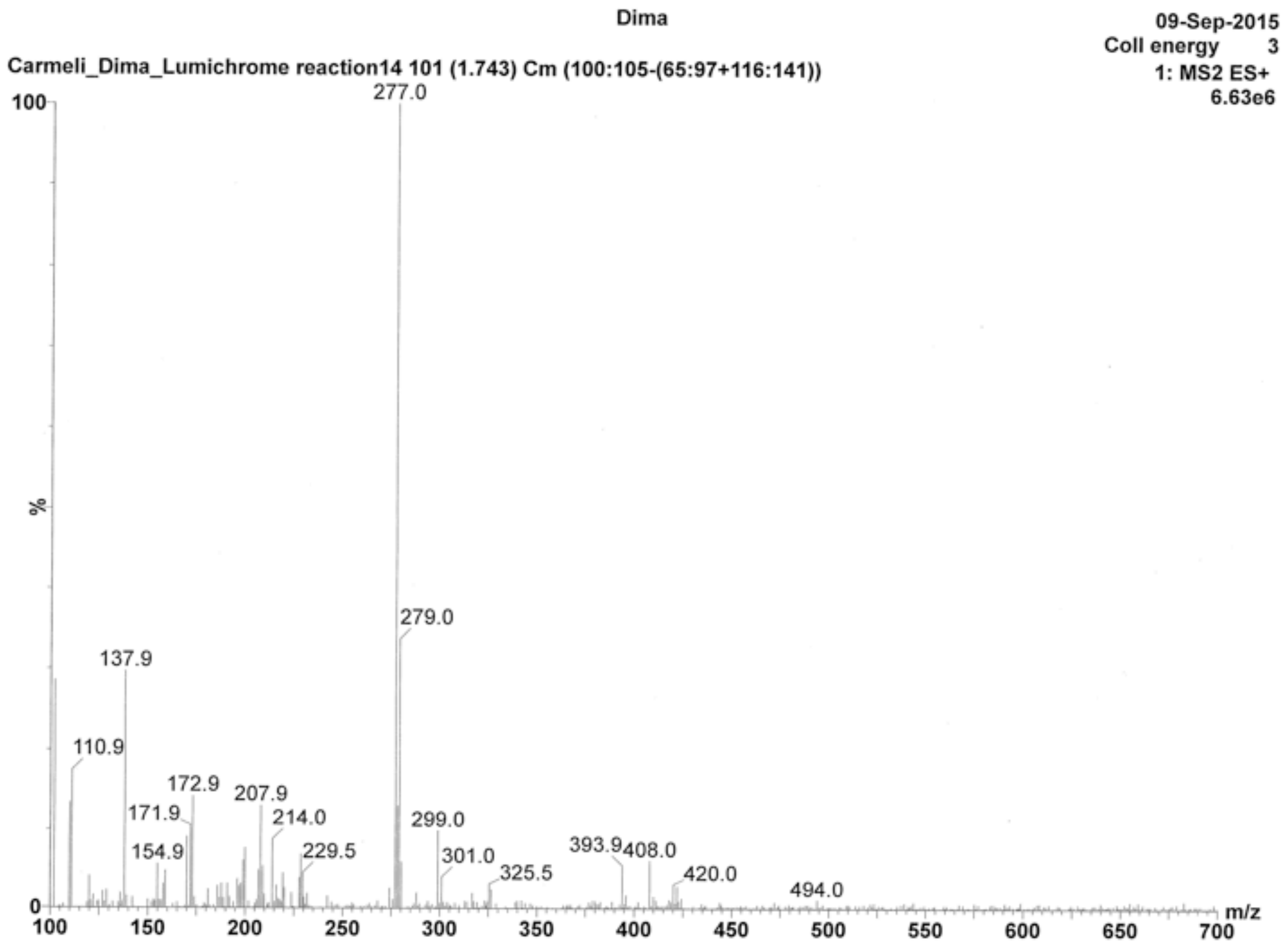

S11. Crystal Structure Report and Table S1 for synthetic 9-Chlorolumichrome (**1**)

A specimen of $C_{14}H_{17}ClN_4O_4$, approximate dimensions 0.120 mm x 0.220 mm x 0.279 mm, was used for the X-ray crystallographic analysis. The X-ray intensity data were measured.

Table S1: Data collection details for synthetic 9-Chlorolumichrome (1)

Axis	dx/mm	2θ/°	ω/°	φ/°	χ/°	Width/°	Frames	Time/s	Wavelength/Å	Voltage/kV	Current/mA	Temperature/K
Phi	37.152	-24.00	-14.51	-10.29	-46.47	0.50	288	5.00	0.71073	50	0.6	n/a
Omega	37.152	-4.00	-51.50	-229.29	99.25	0.50	67	5.00	0.71073	50	0.6	n/a
Omega	37.152	16.00	11.29	-45.17	-44.68	0.50	103	5.00	0.71073	50	0.6	n/a
Phi	37.152	18.50	11.11	-124.64	36.31	0.50	300	5.00	0.71073	50	0.6	n/a

A total of 758 frames were collected. The total exposure time was 1.05 hours. The frames were integrated with the Bruker SAINT software package using a narrow-frame algorithm. The integration of the data using a monoclinic unit cell yielded a total of 10244 reflections to a maximum θ angle of 26.43° (0.80 Å resolution), of which 3140 were independent (average redundancy 3.262, completeness = 99.6%, R_{int} = 2.90%, R_{sig} = 3.53%) and 2551 (81.24%) were greater than $2\sigma(F^2)$. The final cell constants of a = 9.7076(8) Å, b = 21.6114(19) Å, c = 7.4645(5) Å, β = 102.089(4)°, volume = 1531.3(2) $Å^3$, are based upon the refinement of the XYZ-centroids of 160 reflections above 20 σ(I) with 9.395° < 2θ < 45.88°. Data were corrected for absorption effects using the multi-scan method (SADABS). The ratio of minimum to maximum apparent transmission was 0.905. The calculated minimum and maximum transmission coefficients (based on crystal size) are 0.9270 and 0.9680.

The final anisotropic full-matrix least-squares refinement on F^2 with 219 variables converged at R1 = 3.82%, for the observed data and wR2 = 10.53% for all data. The goodness-of-fit was 1.038. The largest peak in the final difference electron density synthesis was 0.282 $e^-/Å^3$ and the largest hole was -0.234 $e^-/Å^3$ with an RMS deviation of 0.056 $e^-/Å^3$. On the basis of the final model, the calculated density was 1.478 g/cm^3 and F(000), 712 e^-.

S12. Table S2. Sample and crystal data for synthetic 9-Chlorolumichrome (**1**)

**Identification code**	car1	
**Chemical formula**	$C_{14}H_{17}ClN_4O_4$	
**Formula weight**	340.76 g/mol	
**Temperature**	110(2) K	
**Wavelength**	0.71073 Å	
**Crystal size**	0.120 x 0.220 x 0.279 mm	
**Crystal system**	monoclinic	
**Space group**	P 1 21/c 1	
**Unit cell dimensions**	a = 9.7076(8) Å	α = 90°
	b = 21.6114(19) Å	β = 102.089(4)°
	c = 7.4645(5) Å	γ = 90°
**Volume**	1531.3(2) $Å^3$	
**Z**	4	
**Density (calculated)**	1.478 g/cm^3	
**Absorption coefficient**	0.276 mm^{-1}	
**F(000)**	712	

S13. Table S3. Data collection and structure refinement for synthetic 9-Chlorolumichrome (**1**)

**Theta range for data collection**	1.89 to 26.43°	
**Index ranges**	-10<=h<=12, -25<=k<=27, -9<=l<=9	
**Reflections collected**	10244	
**Independent reflections**	3140 [R(int) = 0.0290]	
**Coverage of independent reflections**	99.6%	
**Absorption correction**	multi-scan	
**Max. and min. transmission**	0.9680 and 0.9270	
**Refinement method**	Full-matrix least-squares on F^2	
**Refinement program**	SHELXL-2014/7 (Sheldrick, 2014)	
**Function minimized**	$\Sigma\ w(F_o^2 - F_c^2)^2$	
**Data / restraints / parameters**	3140 / 0 / 219	
**Goodness-of-fit on F^2**	1.038	
**Δ/σ_{max}**	0.001	
**Final R indices**	2551 data; $I>2\sigma(I)$	R1 = 0.0382, wR2 = 0.0975
	all data	R1 = 0.0504, wR2 = 0.1053
**Weighting scheme**	$w=1/[\sigma^2(F_o^2)+(0.0490P)^2+0.7914P]$ where $P=(F_o^2+2F_c^2)/3$	
**Largest diff. peak and hole**	0.282 and -0.234 eÅ^{-3}	
**R.M.S. deviation from mean**	0.056 eÅ^{-3}	

S14. Table S4. Atomic coordinates and equivalent isotropic atomic displacement parameters (Å^2) for synthetic 9-Chlorolumichrome (**1**)

U(eq) is defined as one third of the trace of the orthogonalized U_{ij} tensor.

	**x/a**	**y/b**	**z/c**	**U(eq)**
Cl1	0.99836(5)	0.38647(2)	0.95818(6)	0.02139(15)
O1	0.27328(14)	0.20497(6)	0.17563(19)	0.0239(3)
O2	0.11560(13)	0.06025(6)	0.06577(17)	0.0195(3)
O3	0.68848(13)	0.07226(6)	0.67738(18)	0.0224(3)
O4	0.44238(15)	0.13875(7)	0.43793(19)	0.0254(3)
N1	0.03287(15)	0.15559(7)	0.9780(2)	0.0151(3)
N2	0.70255(15)	0.20098(7)	0.68015(19)	0.0154(3)
N3	0.94721(15)	0.25409(7)	0.90117(19)	0.0153(3)
N4	0.90119(15)	0.06744(7)	0.87362(19)	0.0157(3)
C1	0.2772(2)	0.26968(9)	0.2124(3)	0.0250(5)
C2	0.02245(18)	0.09305(8)	0.9775(2)	0.0148(4)
C3	0.92853(18)	0.19384(8)	0.8834(2)	0.0131(4)
C4	0.80595(18)	0.16716(8)	0.7733(2)	0.0147(4)
C5	0.71752(19)	0.26336(8)	0.6954(2)	0.0153(4)
C6	0.60866(19)	0.30222(9)	0.6025(2)	0.0178(4)
C7	0.6178(2)	0.36502(9)	0.6192(2)	0.0194(4)
C8	0.4977(2)	0.40526(10)	0.5238(3)	0.0253(5)
C9	0.84043(18)	0.28974(9)	0.8071(2)	0.0146(4)
C10	0.84782(19)	0.35509(9)	0.8203(2)	0.0172(4)
C11	0.7409(2)	0.39298(9)	0.7310(2)	0.0185(4)
C12	0.79037(18)	0.09888(9)	0.7665(2)	0.0160(4)
C13	0.7485(2)	0.46227(9)	0.7512(3)	0.0263(5)
C14	0.3742(2)	0.08436(10)	0.4811(3)	0.0259(5)

	x/a	y/b	z/c	U(eq)

S15. Table S5. Atomic coordinates and equivalent isotropic atomic displacement parameters (Å^2) for synthetic 9-Chlorolumichrome (**1**)

Cl1-C10	1.7393(19)	O1-C1	1.424(2)
O1-H1	0.80(2)	O2-C2	1.227(2)
O3-C12	1.215(2)	O4-C14	1.419(2)
O4-H14	0.79(3)	N1-C2	1.355(2)
N1-C3	1.381(2)	N1-H13	0.88
N2-C4	1.316(2)	N2-C5	1.358(2)
N3-C3	1.317(2)	N3-C9	1.363(2)
N4-C12	1.378(2)	N4-C2	1.382(2)
N4-H6	0.88	C1-H2	0.98
C1-H3	0.98	C1-H4	0.98
C3-C4	1.419(2)	C4-C12	1.483(3)
C5-C6	1.413(2)	C5-C9	1.424(2)
C6-C7	1.364(3)	C6-H7	0.95
C7-C11	1.439(3)	C7-C8	1.508(3)
C8-H5	0.98	C8-H9	0.98
C8-H8	0.98	C9-C10	1.417(3)
C10-C11	1.379(3)	C11-C13	1.505(3)
C13-H10	0.98	C13-H11	0.98
C13-H12	0.98	C14-H17	0.98
C14-H16	0.98	C14-H15	0.98

S16. Table S6. Bond angles (°) and molecular structure for synthetic 9-Chlorolumichrome (**1**)

C1-O1-H1	108.9(18)	C14-O4-H14	112.2(19)
C2-N1-C3	123.30(15)	C2-N1-H13	118.4
C3-N1-H13	118.4	C4-N2-C5	116.78(16)
C3-N3-C9	115.67(15)	C12-N4-C2	126.70(16)
C12-N4-H6	116.7	C2-N4-H6	116.7
O1-C1-H2	109.5	O1-C1-H3	109.5
H2-C1-H3	109.5	O1-C1-H4	109.5
H2-C1-H4	109.5	H3-C1-H4	109.5
O2-C2-N1	121.91(16)	O2-C2-N4	121.01(17)
N1-C2-N4	117.08(15)	N3-C3-N1	117.98(16)
N3-C3-C4	122.74(16)	N1-C3-C4	119.28(16)
N2-C4-C3	122.31(17)	N2-C4-C12	118.25(16)
C3-C4-C12	119.42(16)	N2-C5-C6	119.55(16)
N2-C5-C9	120.55(16)	C6-C5-C9	119.88(17)
C7-C6-C5	121.26(18)	C7-C6-H7	119.4
C5-C6-H7	119.4	C6-C7-C11	120.08(17)
C6-C7-C8	120.08(18)	C11-C7-C8	119.83(17)
C7-C8-H5	109.5	C7-C8-H9	109.5
H5-C8-H9	109.5	C7-C8-H8	109.5
H5-C8-H8	109.5	H9-C8-H8	109.5
N3-C9-C10	120.40(16)	N3-C9-C5	121.95(17)
C10-C9-C5	117.64(16)	C11-C10-C9	122.44(17)
C11-C10-Cl1	120.56(15)	C9-C10-Cl1	116.99(14)
C10-C11-C7	118.70(17)	C10-C11-C13	121.75(18)

C7-C11-C13	119.54(17)	O3-C12-N4	122.10(17)
O3-C12-C4	123.74(16)	N4-C12-C4	114.15(15)
C11-C13-H10	109.5	C11-C13-H11	109.5
H10-C13-H11	109.5	C11-C13-H12	109.5
H10-C13-H12	109.5	H11-C13-H12	109.5
O4-C14-H17	109.5	O4-C14-H16	109.5
H17-C14-H16	109.5	O4-C14-H15	109.5
H17-C14-H15	109.5	H16-C14-H15	109.5

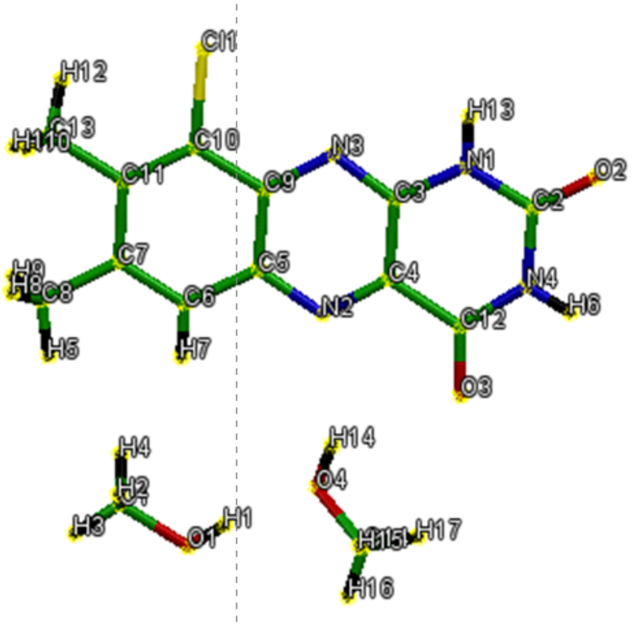

S18. Table S7. Torsion angles (°) for synthetic 9-Chlorolumichrome (**1**)

C3-N1-C2-O2	-178.48(16)	C3-N1-C2-N4	1.1(3)
C12-N4-C2-O2	-178.84(16)	C12-N4-C2-N1	1.6(3)
C9-N3-C3-N1	-179.15(15)	C9-N3-C3-C4	0.3(2)
C2-N1-C3-N3	176.70(16)	C2-N1-C3-C4	-2.7(3)
C5-N2-C4-C3	-0.6(2)	C5-N2-C4-C12	177.33(15)
N3-C3-C4-N2	0.4(3)	N1-C3-C4-N2	179.82(15)
N3-C3-C4-C12	-177.52(15)	N1-C3-C4-C12	1.9(2)
C4-N2-C5-C6	-178.42(15)	C4-N2-C5-C9	0.2(2)
N2-C5-C6-C7	177.83(16)	C9-C5-C6-C7	-0.8(3)
C5-C6-C7-C11	0.9(3)	C5-C6-C7-C8	-177.81(16)
C3-N3-C9-C10	178.33(16)	C3-N3-C9-C5	-0.7(2)
N2-C5-C9-N3	0.5(3)	C6-C5-C9-N3	179.10(16)
N2-C5-C9-C10	-178.57(16)	C6-C5-C9-C10	0.1(2)
N3-C9-C10-C11	-178.43(16)	C5-C9-C10-C11	0.6(3)
N3-C9-C10-Cl1	0.7(2)	C5-C9-C10-Cl1	179.71(12)
C9-C10-C11-C7	-0.6(3)	Cl1-C10-C11-C7	-179.62(13)
C9-C10-C11-C13	178.05(17)	Cl1-C10-C11-C13	-1.0(2)
C6-C7-C11-C10	-0.2(3)	C8-C7-C11-C10	178.50(17)
C6-C7-C11-C13	-178.85(17)	C8-C7-C11-C13	-0.1(3)
C2-N4-C12-O3	178.90(17)	C2-N4-C12-C4	-2.3(3)
N2-C4-C12-O3	1.2(3)	C3-C4-C12-O3	179.26(17)
N2-C4-C12-N4	-177.59(15)	C3-C4-C12-N4	0.4(2)

S19. Table S8. Anisotropic atomic displacement parameters (Å^2) for synthetic 9-Chlorolumichrome (**1**)

The anisotropic atomic displacement factor exponent takes the form: $-2\pi^2[h^2 a^{*2} U_{11} + ... + 2 h k a^* b^* U_{12}]$

	**U11**	**U22**	**U33**	**U23**	**U13**	**U12**
Cl1	0.0230(3)	0.0137(3)	0.0268(3)	-0.00094(18)	0.00365(19)	-0.00206(18)
O1	0.0226(7)	0.0158(7)	0.0279(8)	0.0028(6)	-0.0075(6)	-0.0016(6)
O2	0.0186(7)	0.0117(7)	0.0249(7)	0.0008(5)	-0.0025(5)	0.0024(5)
O3	0.0187(7)	0.0167(7)	0.0285(7)	-0.0020(6)	-0.0029(6)	-0.0020(5)
O4	0.0208(7)	0.0255(8)	0.0261(8)	0.0056(6)	-0.0039(6)	-0.0032(6)
N1	0.0137(7)	0.0111(8)	0.0188(8)	-0.0001(6)	-0.0007(6)	-0.0012(6)
N2	0.0167(7)	0.0145(8)	0.0154(7)	0.0007(6)	0.0043(6)	0.0016(6)
N3	0.0173(8)	0.0126(8)	0.0168(8)	0.0012(6)	0.0054(6)	0.0015(6)
N4	0.0179(8)	0.0079(8)	0.0201(8)	-0.0002(6)	0.0014(6)	0.0002(6)
C1	0.0279(11)	0.0201(11)	0.0249(10)	-0.0022(8)	0.0007(8)	-0.0001(8)
C2	0.0156(9)	0.0138(10)	0.0150(8)	-0.0004(7)	0.0031(7)	0.0001(7)
C3	0.0138(8)	0.0127(9)	0.0142(8)	0.0010(7)	0.0059(7)	0.0021(7)
C4	0.0133(8)	0.0162(10)	0.0153(8)	0.0003(7)	0.0047(7)	0.0009(7)
C5	0.0180(9)	0.0160(10)	0.0141(8)	0.0018(7)	0.0082(7)	0.0024(7)
C6	0.0186(9)	0.0182(10)	0.0169(9)	0.0023(7)	0.0046(7)	0.0029(7)
C7	0.0224(10)	0.0207(11)	0.0173(9)	0.0044(8)	0.0088(8)	0.0063(8)
C8	0.0271(11)	0.0207(11)	0.0277(11)	0.0067(8)	0.0048(9)	0.0079(8)
C9	0.0175(9)	0.0137(9)	0.0144(8)	0.0018(7)	0.0074(7)	0.0018(7)
C10	0.0202(9)	0.0165(10)	0.0165(9)	-0.0002(7)	0.0073(7)	0.0008(8)
C11	0.0256(10)	0.0154(10)	0.0176(9)	0.0029(7)	0.0118(8)	0.0045(8)
C12	0.0145(9)	0.0154(10)	0.0185(9)	0.0004(7)	0.0043(7)	0.0003(7)
C13	0.0364(12)	0.0158(11)	0.0276(10)	0.0029(8)	0.0089(9)	0.0061(9)
C14	0.0227(10)	0.0229(11)	0.0291(11)	0.0028(9)	-0.0011(8)	-0.0012(8)

S20. Table S9. Hydrogen atomic coordinates and isotropic atomic displacement parameters (Å^2) for synthetic 9-Chlorolumichrome (**1**)

	**x/a**	**y/b**	**z/c**	**U(eq)**
H1	0.328(3)	0.1879(12)	0.255(3)	0.036
H14	0.516(3)	0.1440(12)	0.505(4)	0.038
H13	1.1100	0.1727	1.0417	0.018
H6	0.8940	0.0269	0.8762	0.019
H2	0.2663	0.2767	0.3384	0.037
H3	0.2003	0.2902	0.1271	0.037
H4	0.3676	0.2867	0.1970	0.037
H7	0.5275	0.2843	0.5269	0.021
H5	0.4204	0.3790	0.4601	0.038
H9	0.5301	0.4320	0.4349	0.038
H8	0.4645	0.4309	0.6145	0.038
H10	0.6861	0.4758	0.8313	0.039
H11	0.7187	0.4816	0.6305	0.039
H12	0.8455	0.4746	0.8049	0.039
H17	0.4392	0.0492	0.4901	0.039
H16	0.2909	0.0763	0.3845	0.039
H15	0.3455	0.0900	0.5983	0.039

S21. Figure S11. ^{1}H NMR spectrum of veronimide (**2**) in DMSO-d_6

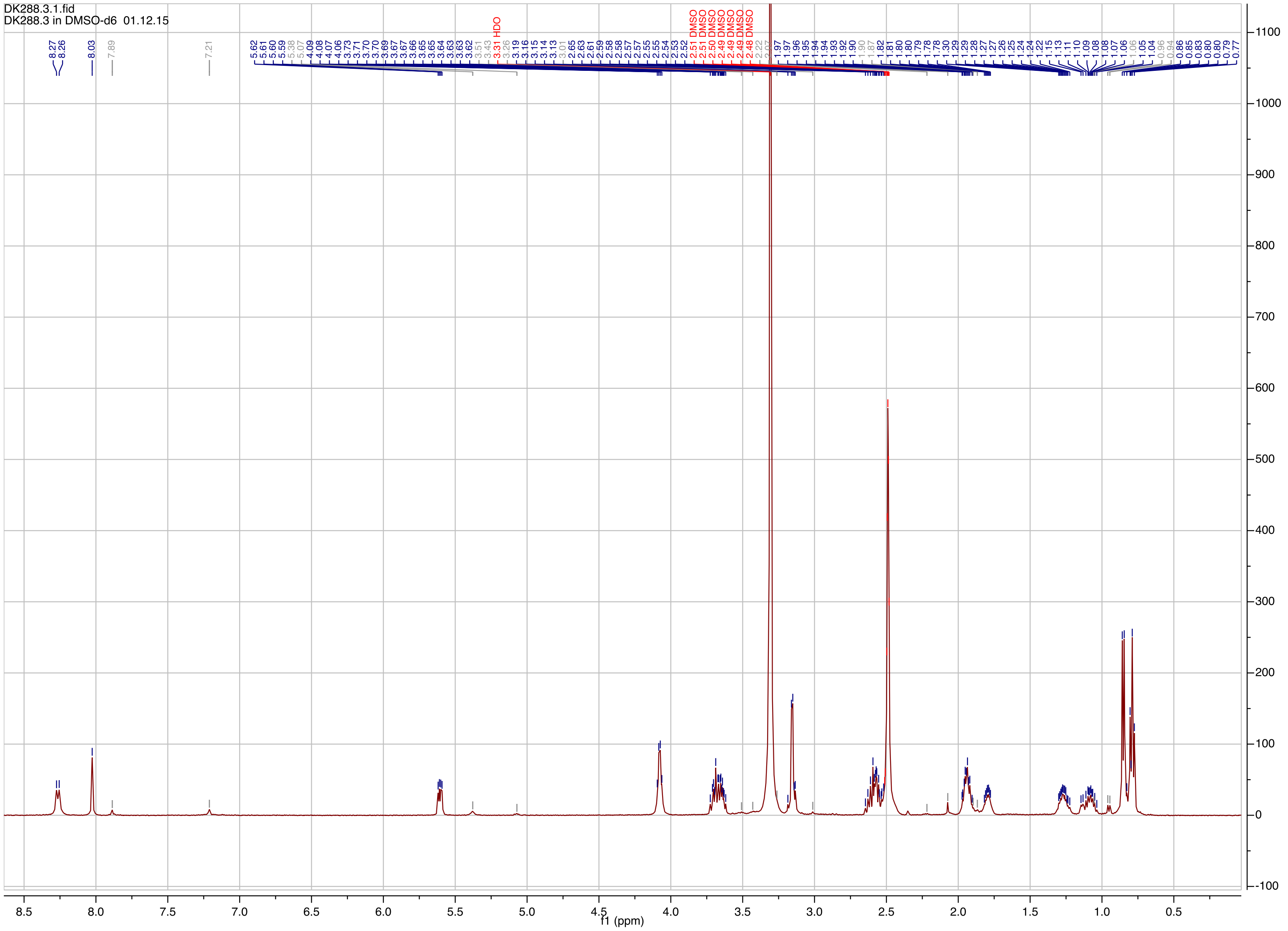

S22. Figure S12. ^{13}C NMR spectrum of veronimide (**2**) in DMSO-d_6

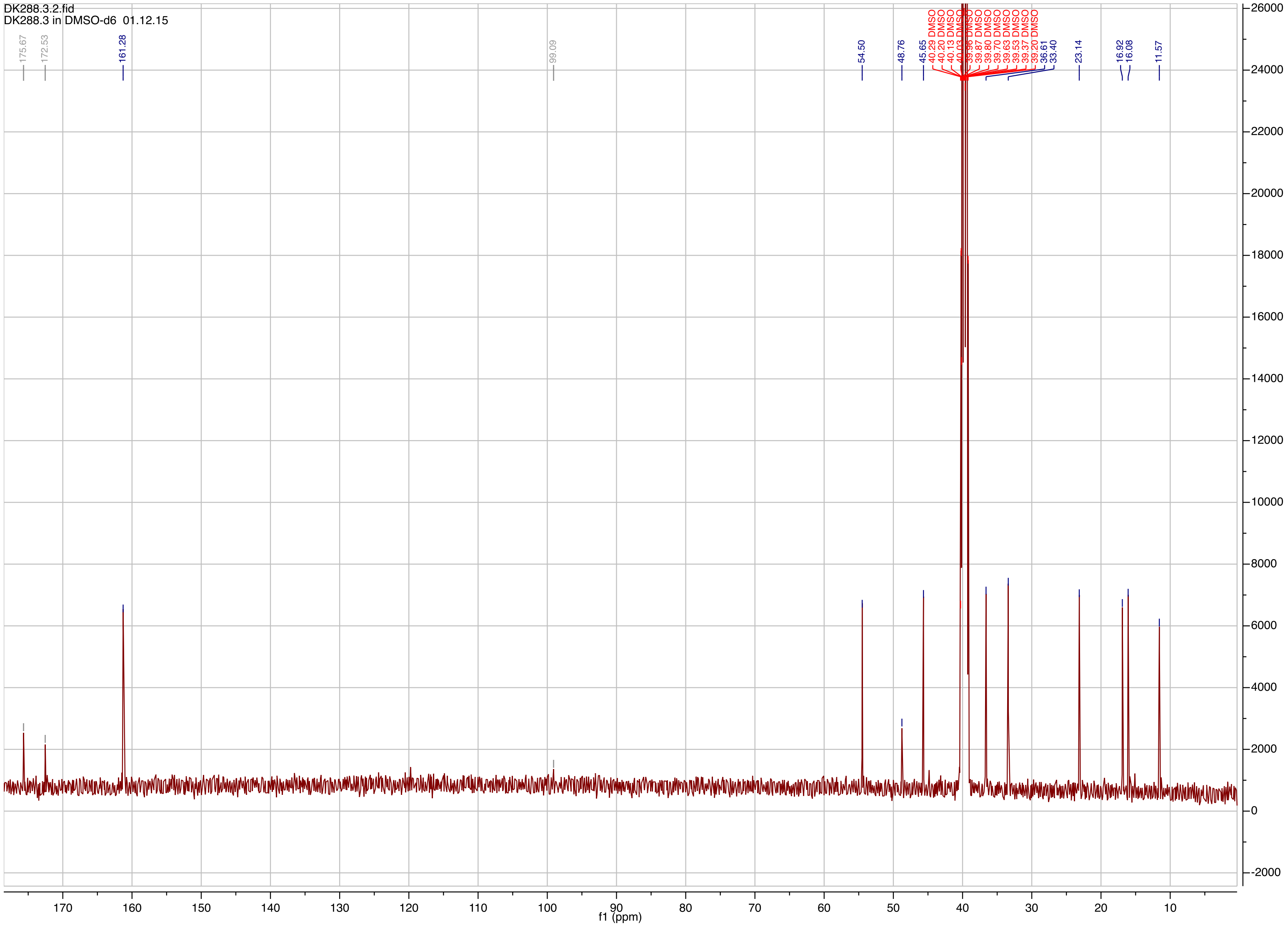

S23. Figure S13. HSQC spectrum of veronimide (**2**) in DMSO-d_6

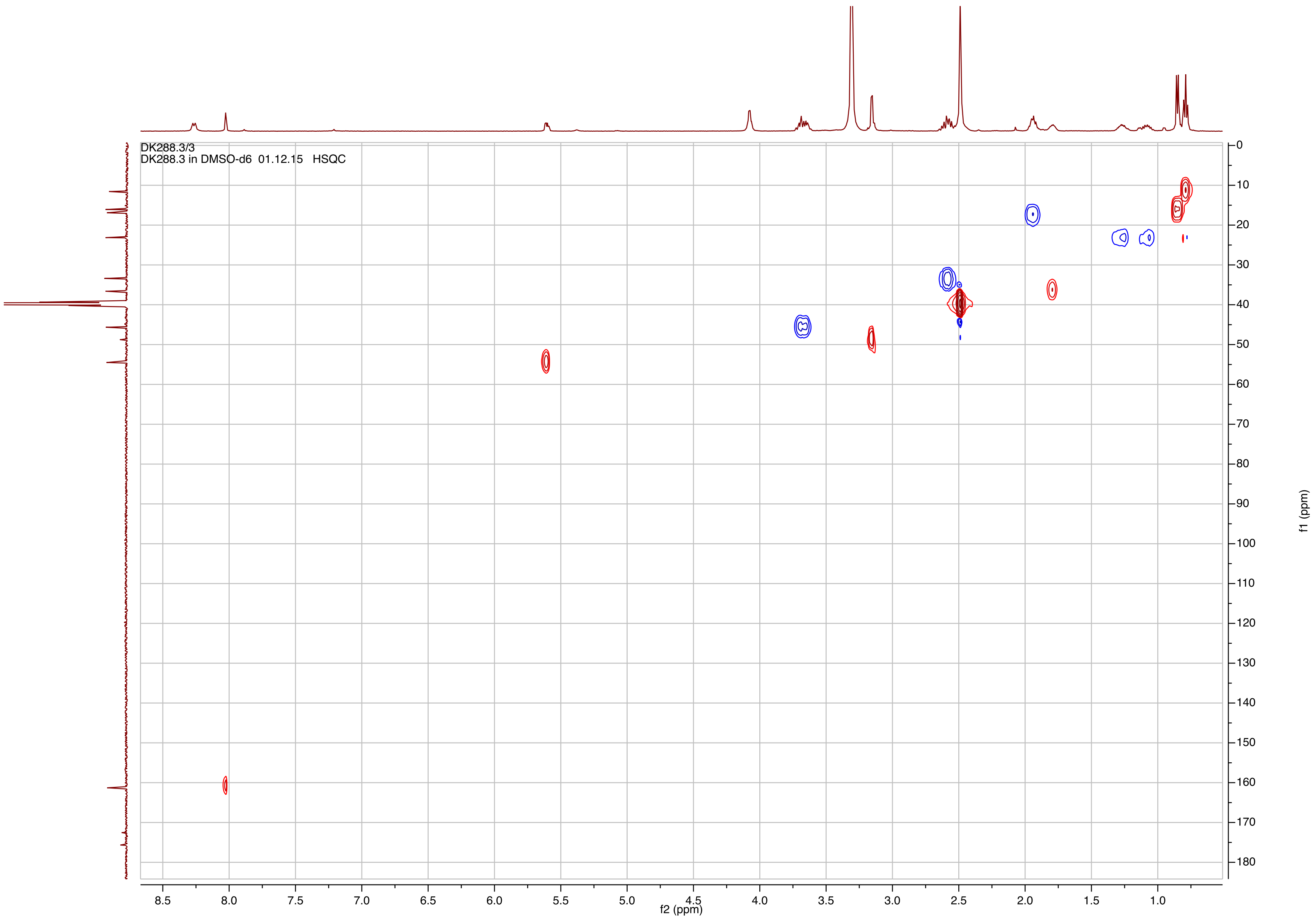

S24. Figure S14. HMBC spectrum of veronimide (**2**) in DMSO-d_6

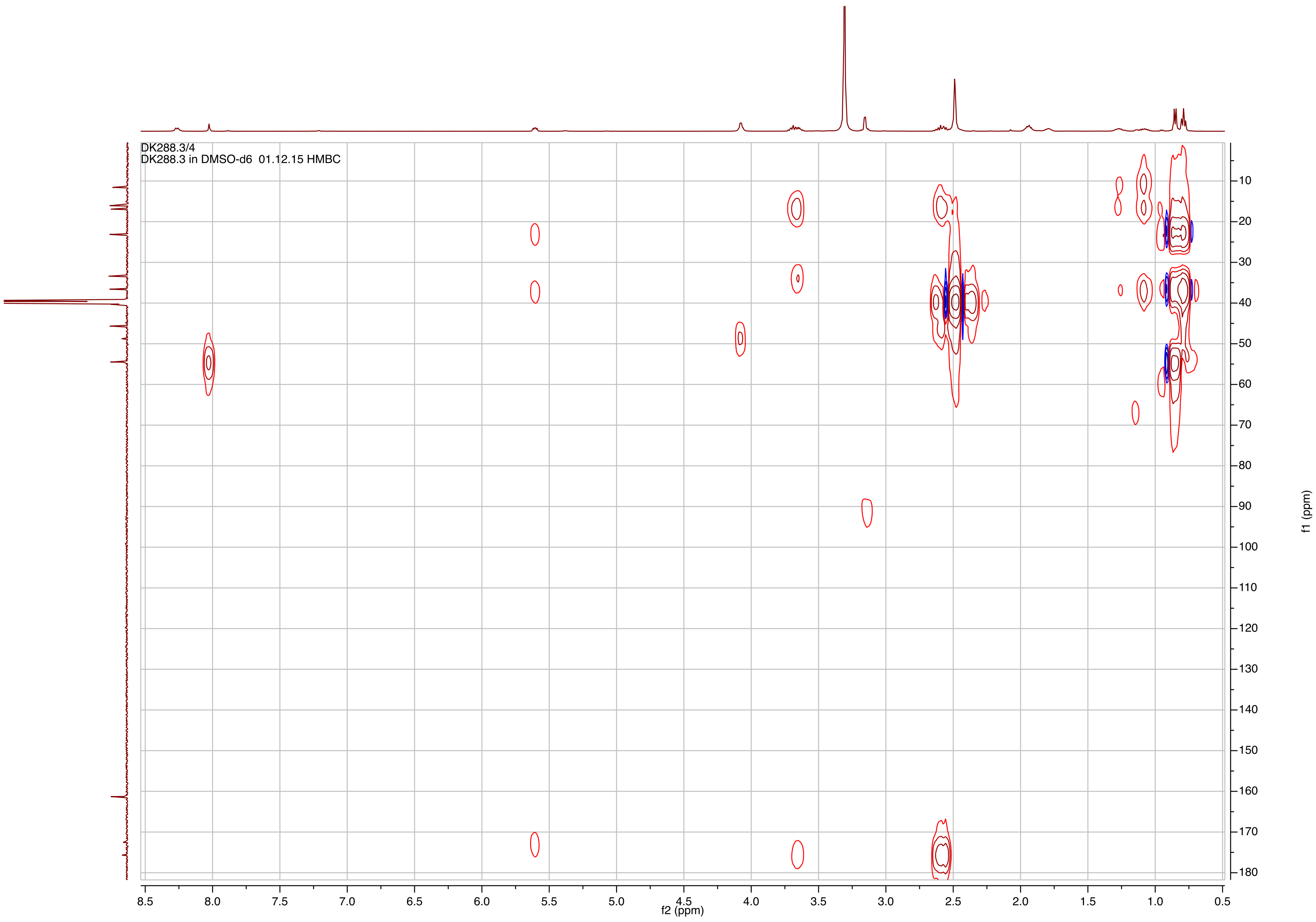

S25. Figure S15. COSY spectrum of veronimide (**2**) in DMSO-d_6

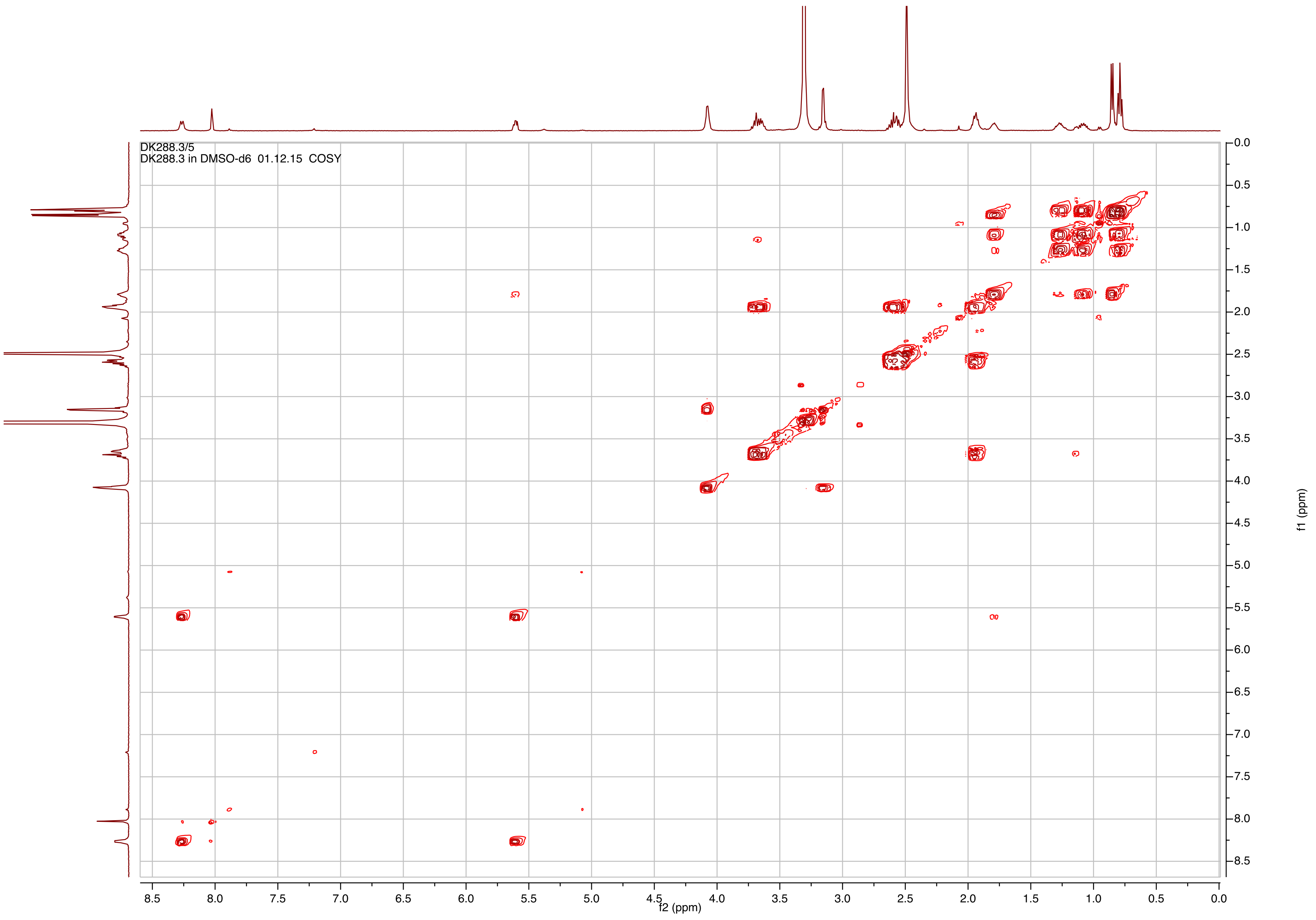

S26. Figure S16. HRESIMS of veronimide (**2**)

Elemental Composition Report

Single Mass Analysis

Tolerance = 2.0 mDa / DBE: min = -1.5, max = 50.0
Element prediction: Off
Number of isotope peaks used for i-FIT = 3

Monoisotopic Mass, Even Electron Ions
309 formula(e) evaluated with 2 results within limits (all results (up to 1000) for each mass)
Elements Used:
C: 0-20 H: 0-30 N: 0-5 O: 0-10 Na: 0-1

1: TOF MS ES+
CARMELI1139 6 (0.280) Cm (5:6)

Dima Kovalerchik

2.00e+004

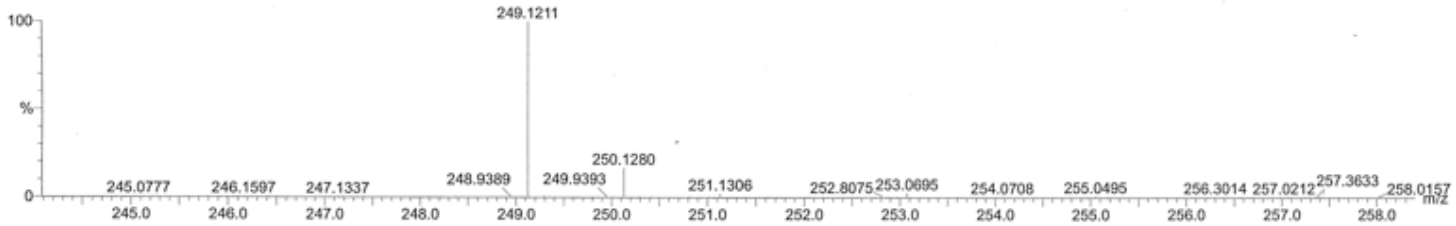

Mass	Calc. Mass	mDa	PPM	DBE	i-FIT	i-FIT (Norm)	Formula
Minimum:				-1.5			
Maximum:		2.0	8000.0	50.0			
249.1211	249.1215	-0.4	-1.6	3.5	328.5	0.1	C11 H18 N2 O3 Na
	249.1199	1.2	4.8	2.5	331.1	2.7	C8 H17 N4 O5

S27. Figure S17. ^{1}H NMR spectrum of veronipyrazine (**3**) in DMSO-d_6

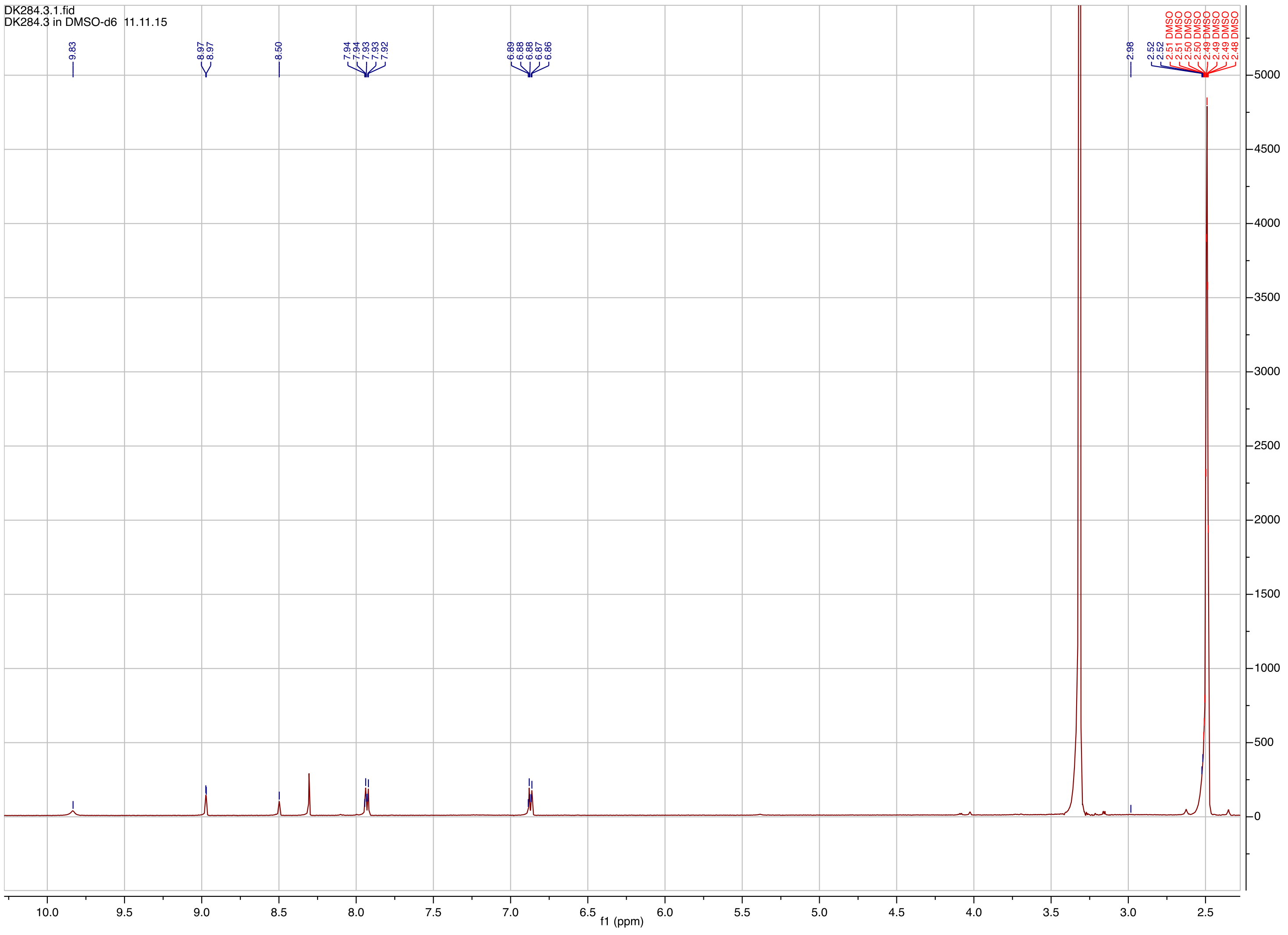

S28. Figure S18. ^{13}C NMR spectrum of veronipyrazine (**3**) in DMSO-d_6

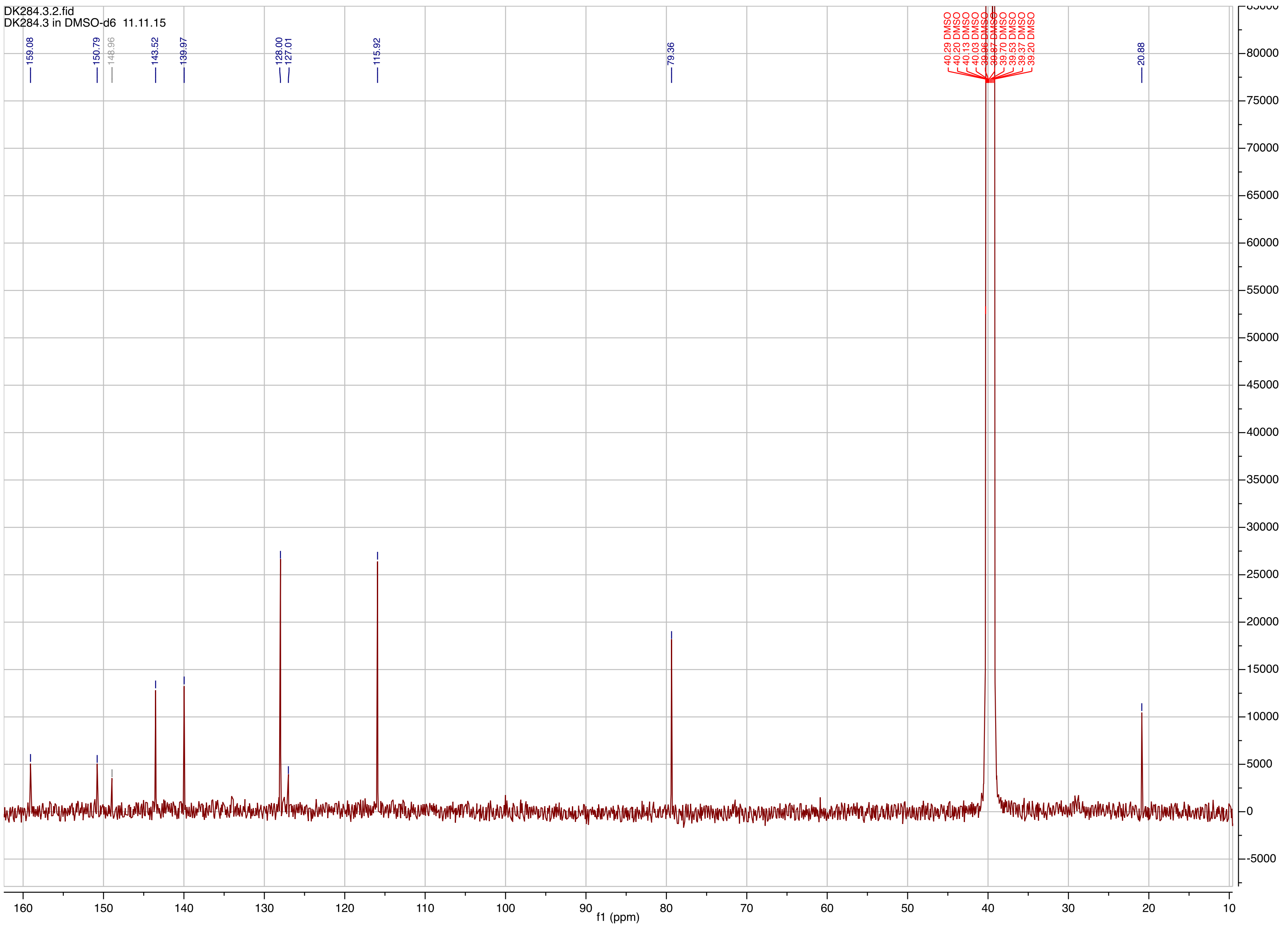

S29. Figure S19. HSQC spectrum of veronipyrazine (**3**) in DMSO-d_6

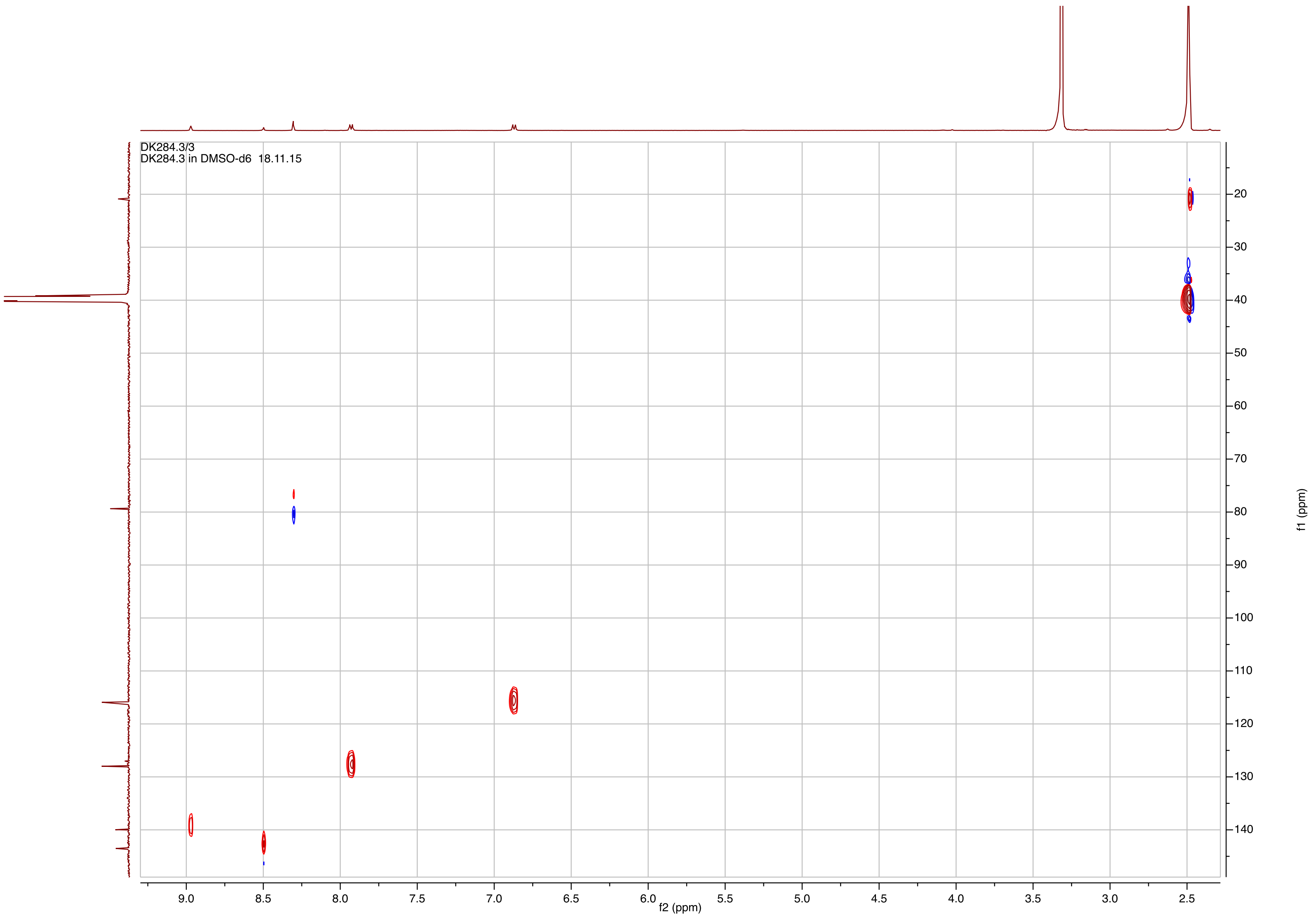

S30. Figure S20. HMBC spectrum of veronipyrazine (**3**) in DMSO-d_6

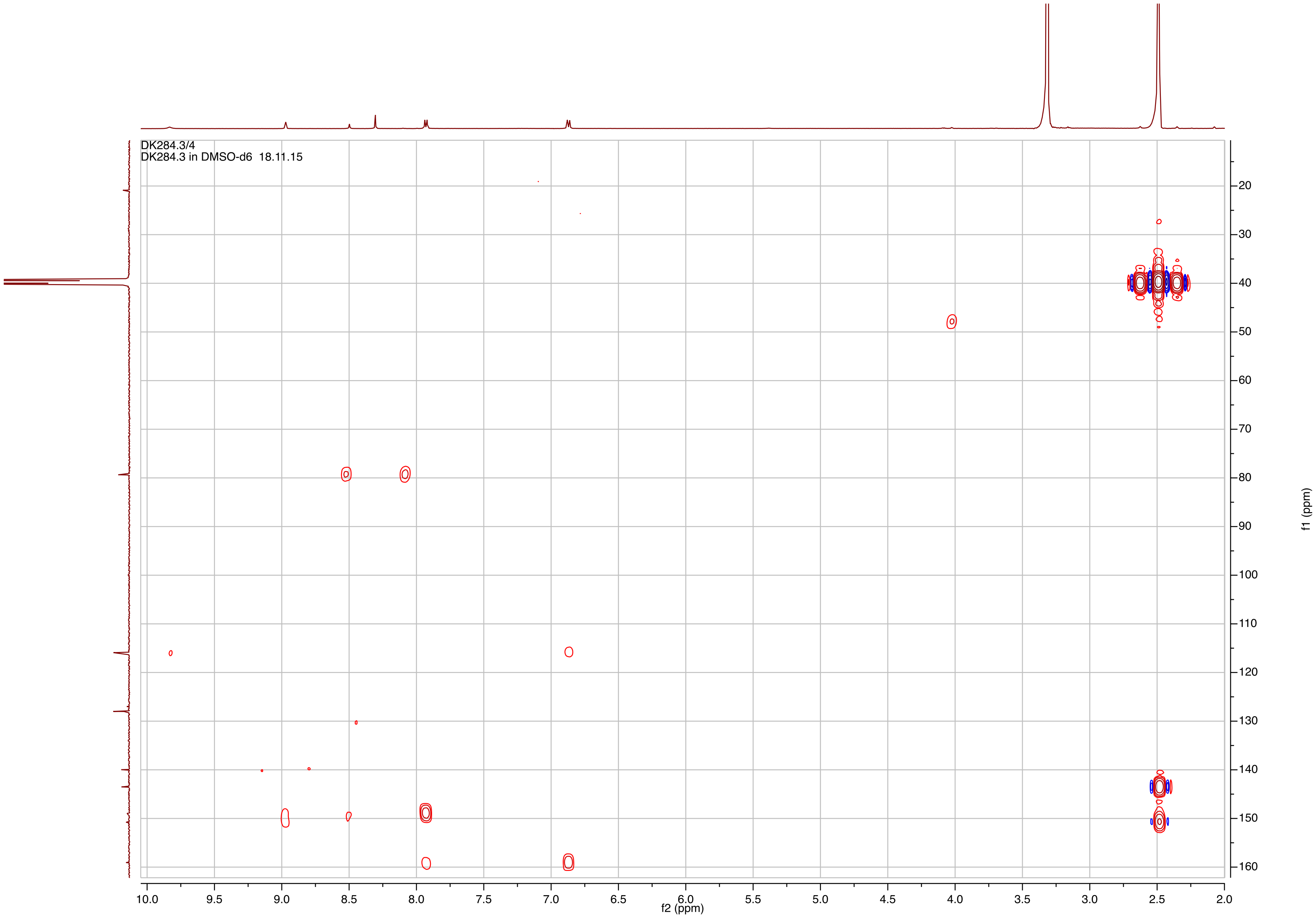

S31. Figure S21. COSY spectrum of veronipyrazine (**3**) in DMSO-d_6

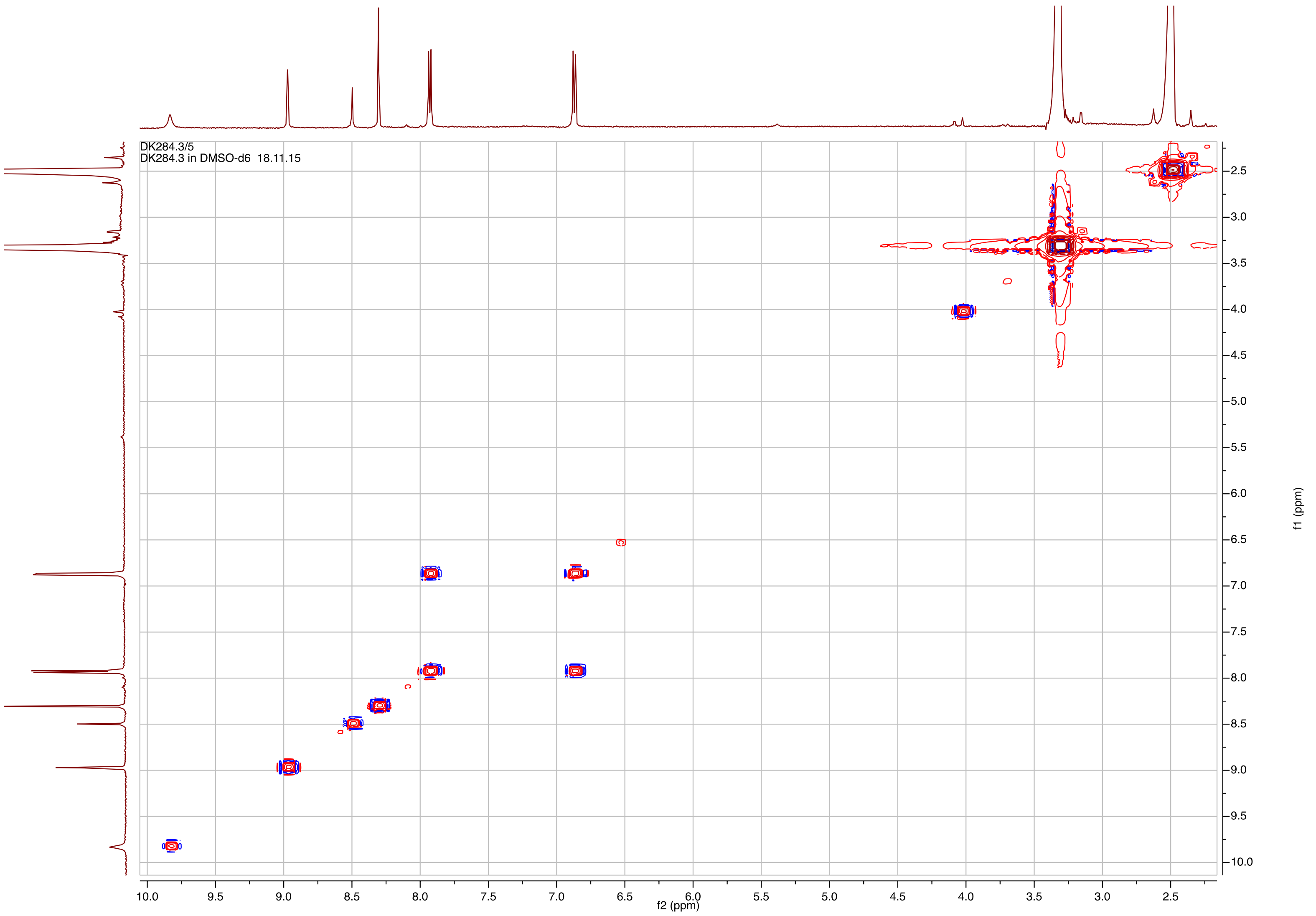

S32. Figure S22. HRESIMS of veronipyrazine (**3**)

Elemental Composition Report

Page 1

Single Mass Analysis

Tolerance = 10.0 mDa / DBE: min = -1.5, max = 50.0
Element prediction: Off
Number of isotope peaks used for i-FIT = 3

Monoisotopic Mass, Even Electron Ions
265 formula(e) evaluated with 13 results within limits (up to 5 best isotopic matches for each mass)
Elements Used:
C: 1-20 H: 1-40 N: 0-10 O: 0-10 Na: 0-1
1: TOF MS ES-
CARMELI1135b 184 (7.000) Cm (184:188)

Dima Kovalerchik

2.55e+004

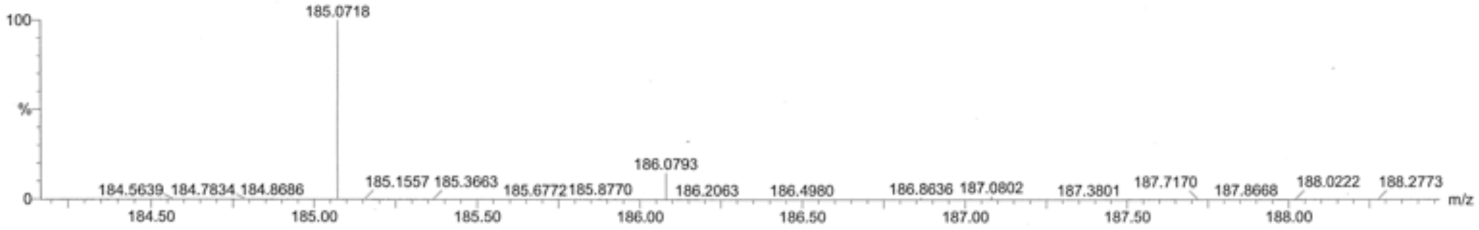

Minimum:				-1.5			
Maximum:		10.0	8000.0	50.0			
Mass	Calc. Mass	mDa	PPM	DBE	i-FIT	i-FIT (Norm)	Formula
185.0718	185.0715	0.3	1.6	8.5	194.5	0.2	C11 H9 N2 O
	185.0691	2.7	14.6	5.5	197.4	3.0	C9 H10 N2 O Na
	185.0790	-7.2	-38.9	0.5	198.7	4.4	C7 H14 O4 Na
	185.0803	-8.5	-45.9	5.5	198.5	4.2	C8 H10 N4 Na
	185.0814	-9.6	-51.9	3.5	197.0	2.7	C9 H13 O4

S33. Figure S23. ^1H NMR spectrum of indole-3-glyoxylamide in DMSO-d_6

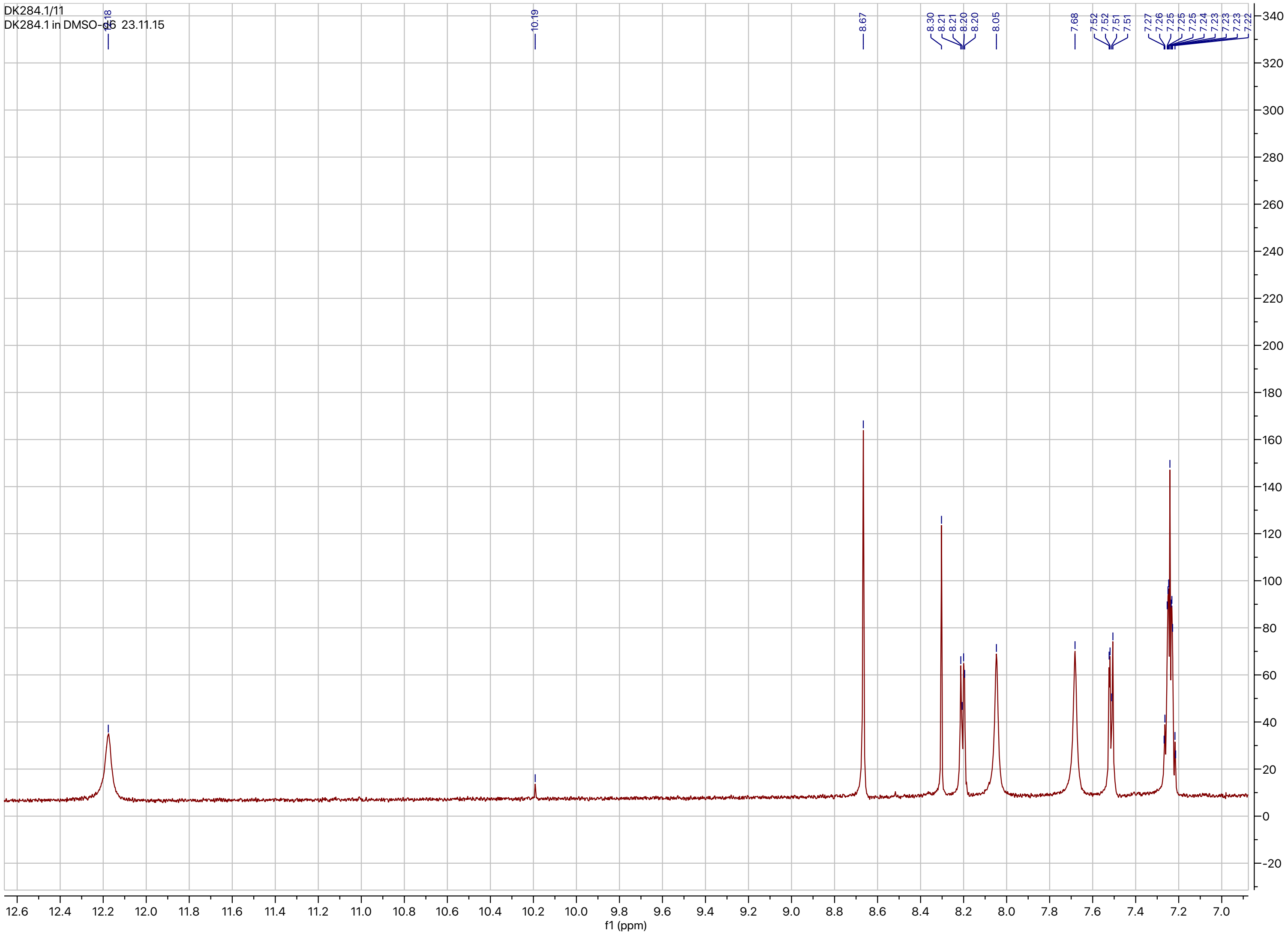

S34. Figure S24. ^{13}C NMR spectrum of indole-3-glyoxylamide in DMSO-d_6

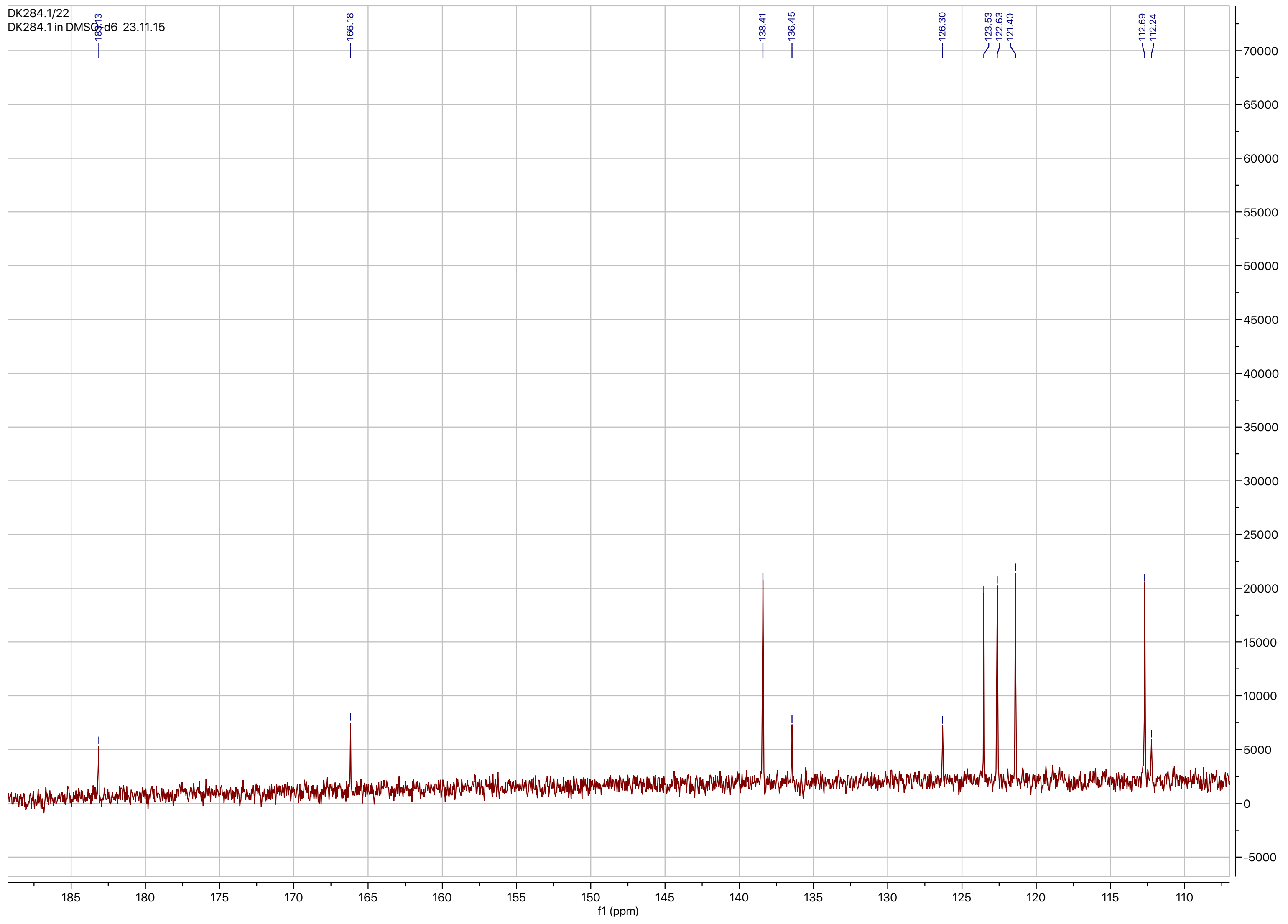

S35. Figure S25. HSQC spectrum of indole-3-glyoxylamide in DMSO-d_6

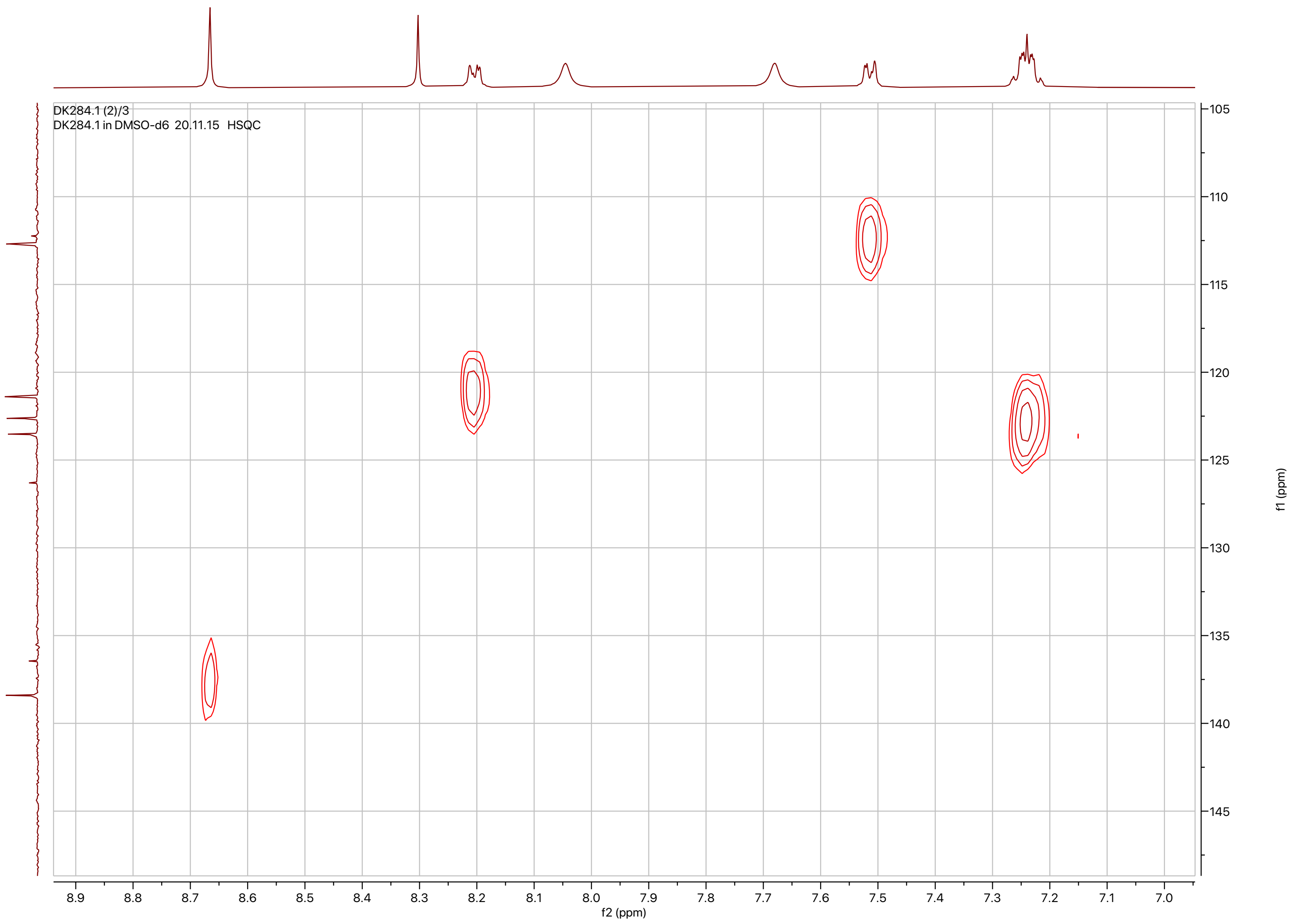

S36. Figure S26. HMBC spectrum of indole-3-glyoxylamide) in DMSO-d_6

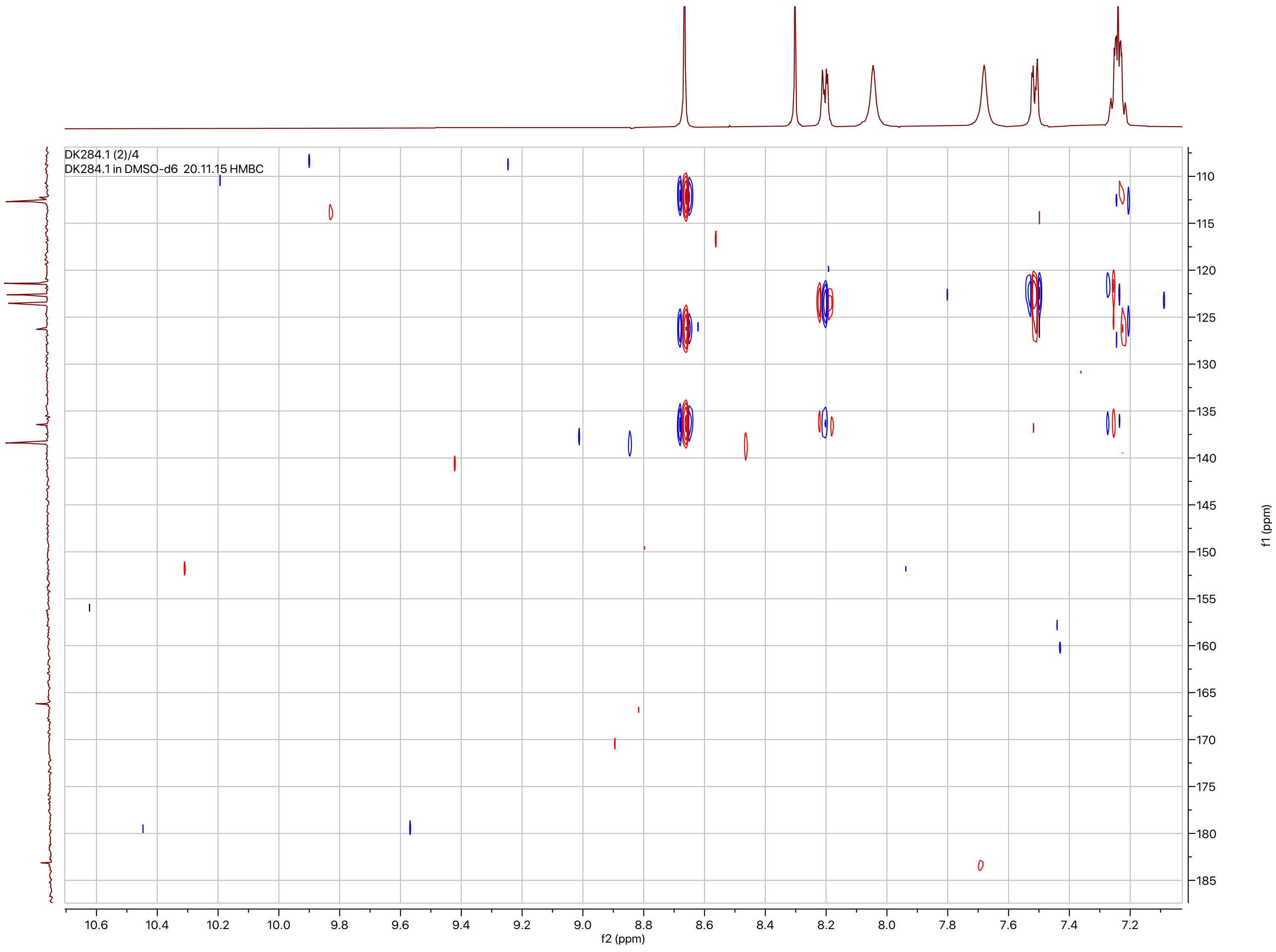

S37. Figure S27. COSY spectrum of indole-3-glyoxylamide in DMSO-d_6

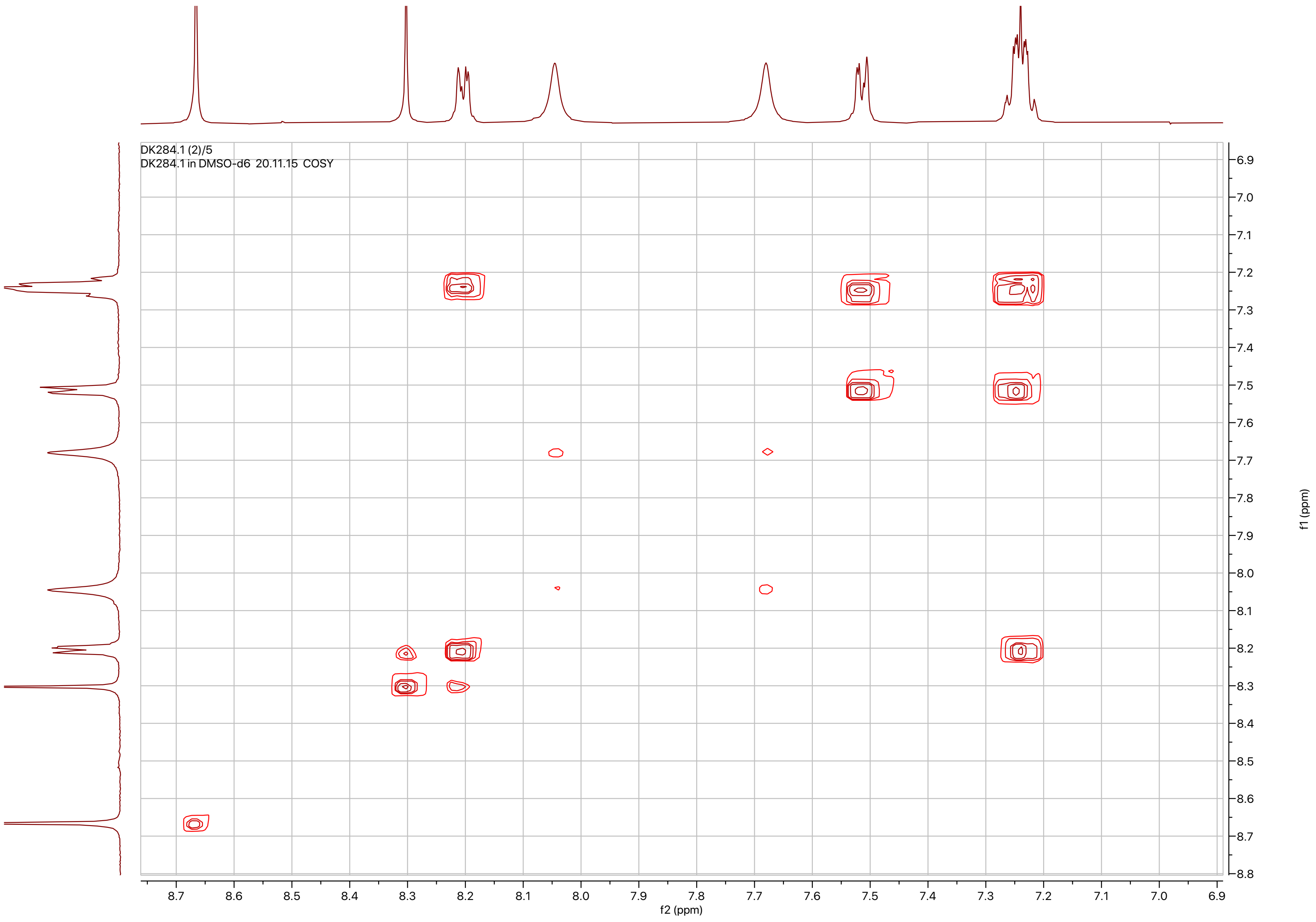

S38. Figure S28. ESIMS of indole-3-glyoxylamide

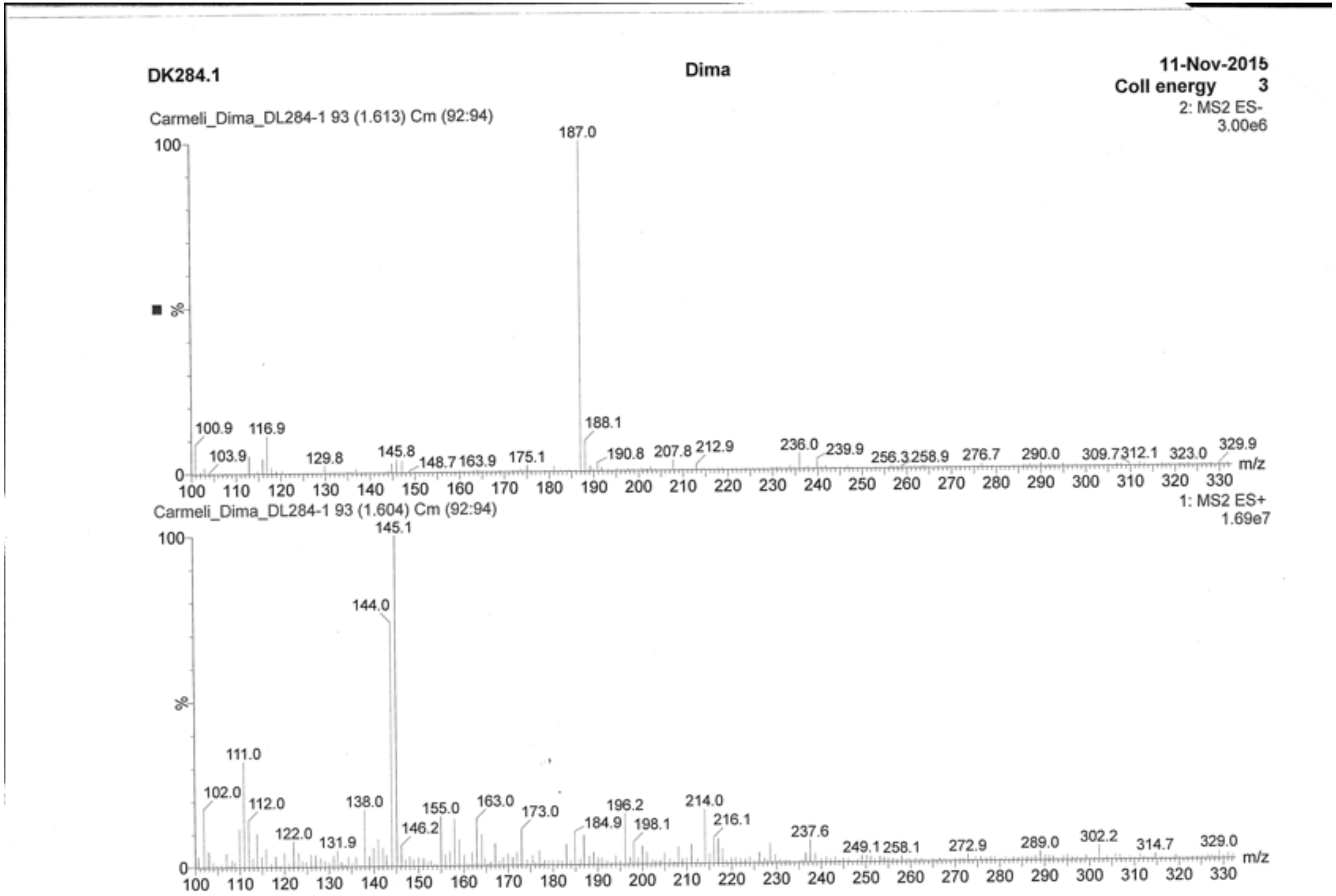

Supplement: Supplementary file 1 [file metabolites-09-00110-s001.pdf]
